# The poor homology stringency in the heteroduplex allows strand exchange to incorporate desirable mismatches without sacrificing recognition *in vivo*

**DOI:** 10.1093/nar/gkv610

**Published:** 2015-06-18

**Authors:** Claudia Danilowicz, Darren Yang, Craig Kelley, Chantal Prévost, Mara Prentiss

**Affiliations:** 1 Department of Physics, Harvard University, Cambridge, MA 02138, USA; 2 School of Engineering and Applied Sciences, Harvard University, Cambridge, MA 02138, USA; 3 Laboratoire de Biochimie Théorique, CNRS UPR 9080, Univ. Paris Diderot, Sorbonne Paris Cité, IBPC, Paris, France

## Abstract

RecA family proteins are responsible for homology search and strand exchange. In bacteria, homology search begins after RecA binds an initiating single-stranded DNA (ssDNA) in the primary DNA-binding site, forming the presynaptic filament. Once the filament is formed, it interrogates double-stranded DNA (dsDNA). During the interrogation, bases in the dsDNA attempt to form Watson–Crick bonds with the corresponding bases in the initiating strand. Mismatch dependent instability in the base pairing in the heteroduplex strand exchange product could provide stringent recognition; however, we present experimental and theoretical results suggesting that the heteroduplex stability is insensitive to mismatches. We also present data suggesting that an initial homology test of 8 contiguous bases rejects most interactions containing more than 1/8 mismatches without forming a detectable 20 bp product. We propose that, *in vivo*, the sparsity of accidental sequence matches allows an initial 8 bp test to rapidly reject almost all non-homologous sequences. We speculate that once the initial test is passed, the mismatch insensitive binding in the heteroduplex allows short mismatched regions to be incorporated in otherwise homologous strand exchange products even though sequences with less homology are eventually rejected.

## INTRODUCTION

RecA family proteins play important roles in DNA recombination and repair. The proteins include RecA itself, which promotes strand exchange in bacteria, as well as Rad51, which promotes strand exchange in eukaryotes. At the beginning of a repair or recombination process, a region of single-stranded DNA (ssDNA) is formed at a DNA double-strand break. The single-stranded region, referred to as the initiating strand, is covered by a recombinase protein forming a helical filament with 6 RecA molecules per helical turn (1). This filament searches a genome for sequence homology by rapidly binding and unbinding double-stranded DNA (dsDNA) until homology is found. If the dsDNA is homologous, the two strands in the dsDNA are known as the outgoing and complementary strands. The initial binding of dsDNA to the recombinase-ssDNA filament is homology independent (2). The binding does not become homology dependent until homology testing begins. Thus, early binding stages will be the same regardless of whether or not the dsDNA is homologous to the ssDNA.

It is believed that during homology testing, the dsDNA binds to the secondary binding site in RecA (3–10). After that binding has occurred, the bases in the complementary strand flip out of the duplex allowing homology to be tested (11). If the sequence of the complementary strand is homologous to the sequence of the initiating strand, base flipping allows the complementary strand to form Watson–Crick bonds with the initiating strand. This transfer of base pairing yields heteroduplex dsDNA bound to the primary binding site (site I) (12), which leaves the bases in the outgoing strand unpaired and bound to the secondary binding site (site II) (4).

The crystallographic structure of the dsDNA bound to site I, referred to as the RecA-heteroduplex complex, is well known (12). It consists of extended and untwisted dsDNA that is divided into nearly B-form base pair triplets separated by large rises (12). It has been proposed that heteroduplex formation depends strongly on base pairing in the heteroduplex. The proposal arose because the crystal structure of the RecA-heteroduplex complex indicated very little favorable interaction between the complementary strand backbone and the protein. The lack of interaction seemed to imply that Watson–Crick pairing dominates the binding of the complementary strand to the filament (12). If the stability of the complementary strand binding to the filament were dominated by the base pairing in the heteroduplex, then homology recognition could be directly governed by the stability of the binding of the bases in the heteroduplex.

In this work, we present experimental and theoretical studies of the mismatch-dependent instability of base pairing in the RecA-heteroduplex complex when it is formed by adding free ssDNA to a solution containing previously formed presynaptic filaments. We then compare those results to the mismatch-dependent instability of dsDNA formed by protein free annealing of ssDNA as well as the mismatch dependence of the strand exchange reaction. We find that the base pairing in the RecA-heteroduplex complex in site I is stronger and less sensitive to the presence of mismatches than the base pairing in B-form dsDNA. Furthermore, the formation of stable strand exchange products can be more stringent than the base pairing in the RecA-heteroduplex complex. We also show that 20-nt sequences containing 5–14 randomly spaced matched bases do not form detectable strand exchange products. This observation is important because the average number of accidental matches in an interaction between two randomly chosen 20 nt sequences in a bacterial genome is 20/4 = 5, and the probability that such an interaction contains more than 14 accidental matches is < 3×10^−5^. Thus, experimental results presented in this work indicate that the vast majority of 20-nt tests that would occur *in vivo* would not result in the formation of a detectable strand exchange product, suggesting that those interactions are very rapidly rejected. Such swift rejection of almost all interactions *in vivo* could allow RecA-mediated homology recognition to rapidly search a bacterial genome even if some rare sequences required longer to resolve (13). Indeed, our experimental results also show that 20-base long oligonucleotides containing 16 or more homologous bases can form 20-bp strand exchange products that are readily detected; however, strand exchange products containing mismatches reverse strand exchange and unbind from the filament significantly more rapidly than homologous strand exchange products. These results combined with earlier theoretical work (13,14) suggest that, *in vivo*, a kinetic recognition system can promote rapid and stringent formation of stable strand exchange products that may include some degree of biologically useful mismatch tolerance.

## MATERIALS AND METHODS

Oligonucleotides were purchased from Integrated DNA Technologies (IDT). Complete sequences of the oligonucleotides used in this work are shown in Supplementary Tables S1 and S2. Lambda phage DNA samples used for single-molecule experiments were prepared by annealing and ligating biotinylated oligonucleotides.

### FRET measurements

FRET experiments for ssDNA binding to the filament were performed by adding a small aliquot of ssDNA to the filament solution in a quartz cuvette. For ssDNA annealing, a small volume of the rhodamine-labeled sequence (Rho c) was added to each fluorescently labeled sequence. FRET strand exchange experiments were performed by mixing a solution containing the unlabeled filament preparation incubated at 37°C for 10 min in the cuvette with a small aliquot of labeled dsDNA. The detection of the emission of the fluorescein label was verified by using 493-nm excitation during 30 min, and the emission was read at 518 nm. The integration was 0.5 s and the band width 2 nm. The sample was kept at all times at 37°C.

The filament was obtained by mixing each reagent to yield a final preparation of 6 μM in bases of the corresponding oligonucleotide, 2 μM of RecA protein (New England Biolabs), 1 mM ATPγS (Sigma-Aldrich), 0.1 μM SSB (single-stranded binding protein Epicentre) and RecA buffer (70 mM Tris-HCl, 10 mM MgCl_2_ and 5 mM dithiothreitol, pH 7.6). Labeled 20-bp dsDNA was prepared from the annealing of the labeled complementary oligonucleotides by cooling down from 80 to 40°C with 2°C steps equilibrated for 1 min; the emission between 500 and 560 nm was acquired (excitation at 493 nm) at each temperature step. The final concentration of dsDNA once added to the filament preparation was 12 μM in bases.

The accuracy of the FRET measurements for strand exchange was evaluated by performing at least three repetitions for several typical cases such as the complete homologous sequence, an oligonucleotide with three internal mismatches (3i), and another with 15 mismatches (pcDNA3). In addition, repetitions performed throughout several days were also analyzed. In the former case the intra-assay accuracy showed a variation between 2 and 4% whereas the inter-assay variation was 6–8% in all of the cases. Binding of ssDNA oligonucleotides to filaments (site I) and annealing experiments were repeated at least twice for all of the cases yielding a 2% variation.

### Single-molecule experiments

In single-molecule experiments, we used 3′3′ and 3′5′-biotin labeled dsDNA to attach to extravidin molecules adsorbed on a glass surface and superparamagnetic beads (4.5 μm in diameter, Invitrogen). After an aliquot of dsDNA in RecA buffer was mixed with RecA (New England Biolabs), ATPγS, and the beads, the resulting sample was placed in a square micro-cell with cross-section of 0.8 mm containing a round inner capillary, 0.55 mm in diameter and closed at its ends. The inner capillary was modified by adsorption of 1 mg/ml extravidin (Sigma) in PBS (phosphate buffered saline) pH 7.4 overnight at room temperature. After an initial incubation of 4 min, the DNA molecules became tethered between the glass capillary surface and the extravidin-coated beads. The micro-cell was then placed in a magnetic tweezers apparatus consisting of a stack of permanent magnets held at a variable distance from the sample. The 5–200 pN force on the beads is controlled by the distance between the magnets and the micro-cell. The position of each bead is tracked by bead tracking software and recorded as described in previous work (15).

### B-form electrostatic calculations

The calculation was done by using the APBS package (16,17). Hydrogen atoms were added to the crystal structures using PDB 2PQR (18) and charges and radii were assigned according to the CHARMM force ﬁeld parameters (19). The calculation was performed at a temperature of 300 K, solute and solvent dielectric constants of 4 and 80, respectively, and ion concentration and exclusion radius of 0.2 M and 2.0 Å, respectively. APBS output including structures with 3D surface potentials were visualized using VMD (20) and PyMol (Schrödinger, LLC).

## RESULTS

FRET based measurements of the sequence dependence of binding of ssDNA to the presynaptic filament, annealing of B-form DNA, and strand exchange.

### Binding of ssDNA to the presynaptic filament

In order to test the proposal that homology recognition originates in the instability of base pairing of mismatched base pairs in the heteroduplex, we performed Förster resonance energy transfer (FRET) experiments to monitor the formation of a RecA-heteroduplex without strand exchange.

**Figure 1.**
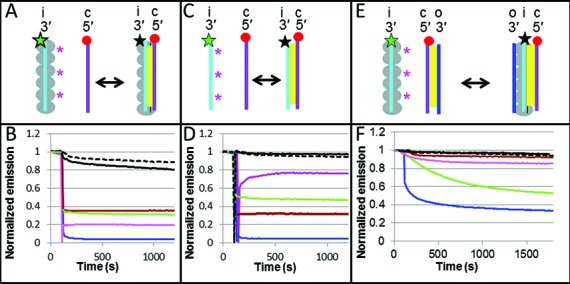
Experiments on 20 nt sequences using the emission of the donor in a FRET pair to study the homology sensitivity of ssDNA binding to the presynaptic filament, free ssDNA annealing and strand exchange. (**A**) Schematic of the binding of free ssDNA with the initiating strand in the presynaptic filament bringing fluorophores in close proximity. The large gray circles indicate RecA molecules. The yellow regions indicate Watson–Crick pairing. The red circle and the star correspond, respectively, to the positions of the rhodamine (acceptor) and fluorescein (donor) FRET partners. The star is green when the fluorescein is emitting and black when it is quenched by the nearby rhodamine. The magenta asterisks indicate the position of the mismatches for a partially matched case. (**B**) Emission of the fluorescein donor versus time for the perfectly matched (blue), mis4 (magenta), 3i (green), 1+2 (brown), pcDNA3 (solid black) and het (dashed black). (**C**) Annealing of free labeled ssDNA molecules without RecA protein. (**D**) Same as (B), except it is for the case shown schematically in panel (C). (**E**) Schematic of the strand exchange experiment. The outgoing, initiating and complementary strands are labeled o, i and c, respectively. (**F**) Fluorescence intensity as a function of time for the strand exchange experiment illustrated in panel (E) and for the same sequences shown in panel (B).

As earlier work showed, dsDNA in the RecA-heteroduplex complex can be obtained without strand exchange using either of two methods: (i) direct dsDNA binding to site I, or (ii) adding complementary ssDNA to the ssDNA-RecA presynaptic filament in which the initiating ssDNA strand is bound to site I (12) (Figure 1A). For the FRET experiments we obtained dsDNA in the RecA-heteroduplex complex by adding free complementary strand ssDNA to the presynaptic filament. In this system, if a free ssDNA binds to the initiating strand in the presynaptic filament, the emission from the fluorescein donor in the initiating strand will decrease because it is quenched by the proximity of the rhodamine acceptor in the complementary strand. Figure 1B shows the measured fluorescence as a function of time for experiments performed at 37°C. The matched strands show practically complete quenching, indicating that almost all of the ssDNA molecules with fully complementary sequence bind to the presynaptic filament and remain bound most of the time. Thus, for the matched sequence the binding rate strongly dominates over the unbinding rate.

It is possible that the binding of free ssDNA to the presynaptic filament could be insensitive to mismatches between the free ssDNA and the initiating strand. For example, the free ssDNA could bind to the secondary binding site without interacting with the initiating strand. If the complementary strand binding to the presynaptic filament were insensitive to mismatches, then the fluorescence signal for the heterologous, homologous and nearly matched ssDNA molecules would all be the same; however, the signals for all of these are markedly different indicating that the binding is sensitive to mismatches.

Though the binding is sensitive to mismatches, it is in general not very stringent. If homology stringency were perfect, the asymptotic fluorescence value should not change when the ssDNA added to the active filament contains mismatches. Instead a small change in fluorescence was detected when the presynaptic filament interacted with an ssDNA sequence that contains no matched bases when aligned in registration with the initiating strand (Figure 1B). The small decrease still corresponds to ∼10% of the change for the homologous sequence. When a 20-nt sequence from pcDNA3 with 5/20 randomly positioned matched bases was combined with the presynaptic filament, the fluorescence continued to decrease throughout the observation time. At the end of the observation period, the fluorescence change corresponded to ∼20% of the change for the homologous sequence. Such significant binding of sequences with only 5/20 matches could pose challenges for homologous recognition *in vivo* where ∼20% of the interactions would contain 5/20 accidental matches. Sequences containing 16 or 17 matched bases show 40 to 60% fluorescence change with respect to the homologous sequence, suggesting that base pairing in the RecA-heteroduplex complex can be quite stable in the presence of three or four mismatches.

Though sequences containing a small number of mismatches can show fairly stable binding, the data clearly indicate that heteroduplexes containing mismatches yield lower equilibrium binding values than the homologous sequence. We note that previous fluorescence studies have also found that the binding of ssDNA in the heteroduplex favors the homologous interaction (21).

### B-form dsDNA obtained by ssDNA annealing

Having evaluated the mismatch-dependent instability provided by the binding of free ssDNA to ssDNA bound to site I, we wanted to compare those results to the mismatch-dependent instability provided by the annealing of free ssDNA to form B-form dsDNA under the same conditions. Thus, we hybridized the same fluorescein and rhodamine labeled ssDNA molecules in the absence of RecA protein, as illustrated in Figure 1C. The resulting fluorescence versus time measurements are shown in Figure 1D. Both the perfectly matched and heterologous oligonucleotides reach equilibrium in ∼100 s. The fluorescence curve for the homologous sequence is nearly identical to the curve for binding free ssDNA to the presynaptic filament; however, for many sequences protein free annealing of ssDNA provides better rejection than binding to the presynaptic filament.

### Strand exchange

In order to study the relationship between the homology stringency provided by the binding of the complementary strand to the presynaptic filament and the stringency provided by strand exchange, we performed additional FRET experiments (Figure 1E). These experiments were designed to measure the homology dependence of the equilibrium binding of strand exchange products, so we could compare the results with those obtained for annealing and ssDNA binding to the presynaptic filament.

Figure 1E shows a schematic representation of strand exchange measurements that are analogous to the experiments shown in Figure 1A and C. For these experiments, the initial dsDNA consists of 20-bp sequences from lambda phage DNA containing the rhodamine fluorophore at the 5′ end of the complementary strand whereas the fluorescein donor is attached to the 3′ end of the initiating strand (presynaptic filament) (Supplementary Table S1). At the beginning of the experiment, the fluorescein in the ssDNA-RecA filament emits brightly. When the rhodamine labeled complementary strand comes close enough to the initiating strand, the fluorescein emission decreases as a result of the quenching due to the rhodamine acceptor (Figure 1F). Thus, if dsDNA binds to the presynaptic filament in a position where the complementary strand can undergo strand exchange, then the fluorescein emission should decrease.

Figure 1F shows curves of the fluorescein emission versus time for strand exchange reactions between 20-bp dsDNA and 20-nucleotide filaments at 37°C in a buffer containing ATPγS, which presents negligible hydrolysis. Since strand exchange progresses through homologous regions at ∼6 bp/s (22–24), strand exchange of 20-nt homologous sequences should complete in less than 4 s. Thus, strand exchange is nearly instantaneous on the timescale of the fluorescence evolution shown in Figure 1F (> 20 min).

In Figure 1F, the blue curve shows the fluorescence for the homologous strand exchange reaction. The curve shows that the fluorescence approaches an asymptotic limit that is ∼35% of the initial fluorescence. In contrast, binding of homologous free ssDNA to the presynaptic filament results in a fluorescence decrease corresponding to ∼95% of the initial fluorescence (Figure 1B). The remaining curves in Figure 1F represent the fluorescein emission due to the strand exchange of sequences containing mismatches. Those curves show that the asymptotic fluorescence limits for mismatched sequences are higher than for the homologous sequence. Those higher asymptotic values indicate that the strand exchange products for the mismatched sequences are less favorable than the product for the homologous sequence. Furthermore, the fluorescence due to a sequence with 5/20 matched bases shows almost no shift, indicating that the complementary strand does not remain bound to the presynaptic filament in a position where it could undergo strand exchange.

### Single-molecule pulling shows that the binding of the complementary strand in the RecA-heteroduplex complex is more stable than the binding between annealed ssDNA strands in B-form dsDNA

The asymptotic values of the fluorescence curves for direct ssDNA binding to an ssDNA-RecA filament indicate that base pairing in the heteroduplex is less stringent than the pairing in B-form dsDNA; however, it does not provide information about the relative stability of the base pairing in the two structures. Crystallographic studies have shown that backbone interactions do not play a significant role in binding the complementary strand to the heteroduplex (12), so the complementary strand must be bound to the heteroduplex via interactions involving the complementary strand bases. Thus, the stability of the binding in the heteroduplex can be probed by single-molecule pulling experiments that apply forces to the DNA backbones resulting in tension on the complementary strand bases. Such experiments can probe the stability of the binding of the complementary strand by measuring the force that must be applied to the ends of the DNA in order to separate the complementary strand from the filament. Increasing the shear force increases the stress on the complementary strand bases binding that strand to the filament until the bonds connecting the complementary strand to the filament are disrupted.

For these experiments, we formed the RecA-heteroduplex complex by directly binding dsDNA to site I. Single molecules are selected by following the overstretching curve initially when there is no significant amount of RecA protein bound to dsDNA. This curve should show that the overstretching transition takes place at around 65 pN; higher forces indicate that there is more than one dsDNA trapped between the bead and the capillary surface, and therefore such cases are not included in our studies. Successive overstretching cycles lead to RecA nucleation on dsDNA which allows complete coverage of the dsDNA molecule by polymerized RecA within one hour (25). Under our experimental conditions there is a significant lag time for RecA nucleation onto dsDNA, so the pulling experiments start with naked dsDNA (25). When dsDNA is fully covered by RecA, the overstretching transition disappears, and for a molecule that is partially covered, only the uncovered dsDNA is able to overstretch.

**Figure 2.**
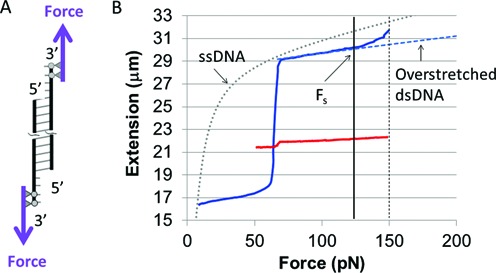
Extension versus force curves for naked dsDNA and dsDNA bound to site I pulled from the 3′3′ ends. (**A**) Schematic illustration of the pulling technique. (**B**) Extension versus force curves. The solid blue and red curves show, respectively, typical results for naked dsDNA (15) and dsDNA bound to site I in an almost complete filament. The dashed blue and dotted gray curves are theoretical plots of the extension versus force curves for overstretched dsDNA and ssDNA, respectively (15). The solid black vertical line at ca. 125 pN indicates F_s_, the force at which naked dsDNA begins to shear and the dashed black line indicates the shearing force ∼150 pN (15).

For the pulling experiment results shown in Figure 2, we monitored the extension versus force curve as the RecA coverage increased. Initially, when the dsDNA was not yet covered by the protein, we observed the usual overstretching transition at 65 pN, which indicated that we were observing one single tethered dsDNA molecule (26). As time went on, the RecA covered the dsDNA. The coverage could be monitored by measuring the increase in extension and the decrease in the fraction of the dsDNA that participates in the overstretching transition. For the dsDNA-RecA filament shown in Figure 2, the coverage of the filament was not quite complete. The remaining overstretching transition at 65 pN shows that the dsDNA being monitored is still one single molecule. Figure 2 also shows the results of pulling experiments for B-form dsDNA. Extensive studies of that system were presented in previous work (15). The curve represents a typical result from that study. Figure 2 indicates that the binding of the complementary strand in the ssDNA-RecA filament blocks the overstretching transition that occurs at ∼65 pN when B-form dsDNA is pulled from the 3′3′ ends, as well as the shearing transition that occurs in B-form dsDNA when the applied force is ∼120 pN. These data indicate that the complementary strand binds more strongly to the ssDNA-RecA filament than it binds to its partner strand in protein-free B-form dsDNA annealing. Supplementary Figure S1 shows similar results for 3′5′ pulling. Previous work has also shown that the binding of RecA to dsDNA blocks the overstretching transition when a single dsDNA molecule is pulled from the 3′5 ′ ends (27).

### MD simulation experiments suggest the complementary strand binding in the RecA-heteroduplex complex can be insensitive to mismatches

**Figure 3.**
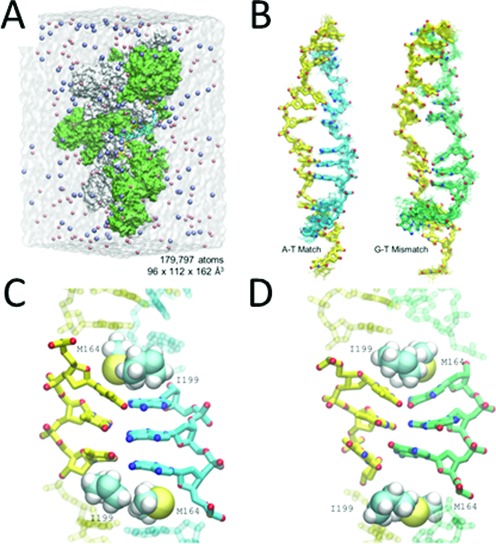
Molecular Dynamics simulation for poly dA-dT and poly dG-dT dsDNA in site I. (**A**) Schematic of the molecular dynamics simulation system. The solvation water is shown as the transparent box and the sodium and chloride ions are shown as pink and purple sphere, respectively. The molecule in surface representation is the RecA-DNA complex. (**B**) Structure of the matched and mismatched DNA in site I at the end of the 15 ns conventional MD simulation. The initiating, and matched and mismatched complementary strands are shown in yellow, cyan and green, respectively. The overlapping structures are obtained from the last 10 ns of the simulation with 1 ns gap. (**C** and **D**). Detail illustrations of the stable nucleotide triplet at the end of the simulation for matched and mismatched strands.

MD simulations offer a third tool for considering the stability of dsDNA binding in site I. Though Figure 1F shows that completely heterologous sequences do not form observable strand exchange products, sequences with just 5/20 matched bases can produce detectable products when ssDNA binds to the presynaptic filament (Figure 1B). In order to probe the stability of highly mismatched sequences in the RecA-heteroduplex, we turned to molecular dynamics (MD) simulations (Figure 3A) that allow either completely matched or completely mismatched sequences to be placed in the heteroduplex. A description of the MD protocol can be found in Supplementary Data. We then compared the time evolution of matched and mismatched heteroduplexes. Figure 3B shows the simulated evolution of the structure of the crystallized poly(A)-poly(T) heteroduplex in the RecA filament structure (12). Figure 3B also shows the analogous simulated evolution for a poly(T)-poly(G) structure. In the poly(T)-poly(G) structure, there is a mismatch at every position in the sequence.

Figure 3 suggests that Watson–Crick pairing can maintain the stability of the positioning of complementary strand bases in the absence of protein residues maintaining intrastrand stacking as discussed in more detail in Supplementary Data; however, Watson–Crick pairing is not required to stabilize the positioning of complementary strand bases over 10 ns timescales if protein residues maintain intrastrand stacking.

### Backbone repulsion in the RecA-heteroduplex complex is smaller than in B-form dsDNA

**Figure 4.**
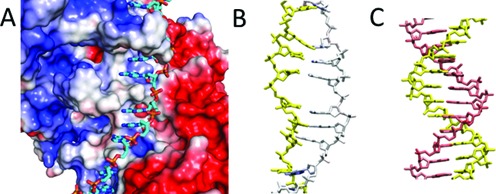
Potential maps of the presynaptic filament and B-DNA. (**A**) Map for the presynaptic filament at the surface of the protein and the initiating strand (both in surface representation), where blue corresponds to +3 kT/e, white is neutral, and red corresponds to −3 kT/e. The potential includes the effects of charges in the initiating strand. The complementary strand (in stick representation) is not present in the presynaptic filament, but it is shown to indicate the position it occupies when dsDNA is bound to site I. The complementary strand coloring represents the atomic content, not the potential. It is positioned in a neutral region separating regions with strong positive and negative potentials (12). (**B**) Same as panel (A), but only the potential at the position of the complementary strand is shown. The protein is not shown, and the initiating strand is shown in yellow in stick representation. The nearly white color in the complementary strand indicates that the potential is almost neutral on this scale. Similar results are obtained for the same conformation of the two DNA strands (stretched-unwound form) in the absence of the protein, as shown in Supplementary Figure S3. (**C**) Calculation for B-form dsDNA analogous to the calculation shown in Supplementary Figure S3, mapping the potential exerted by one (yellow) strand on the complementary strand. The pink color indicates a negative potential.

Results of experiments and simulations indicated that the binding in the RecA-heteroduplex complex is more stable and mismatch insensitive than in B-form dsDNA, but they have not provided any reason for that enhanced stability. We sought possible origins by making potential maps of the RecA-heteroduplex complex like those shown in Figure 4 and Supplementary Figures S2 through S6. Consistent with previous work (12), the electrostatic maps do not show a strong favorable interaction between the complementary strand backbone and the filament; however, comparison between electrostatic maps of the RecA-heteroduplex complex and maps of B-form dsDNA do show some significant differences that might make complementary strand binding in the RecA-heteroduplex complex more stable and mismatch insensitive than in B-form dsDNA. In particular, Figure 4B shows that the initiating strand has little electrostatic influence on the complementary strand, whereas in B-form dsDNA there is a substantial repulsive interaction between the strands (Figure 4C). Finally, Supplementary Figure S2 shows that negatively-charged amino acid residues create a repulsive potential that pushes the complementary strand toward the initiating strand, whereas in B-form dsDNA the interstrand repulsion pushes the two strands away from each other. The force exerts stress on the base pairing that connects the two strands; however, that stress is not present in the RecA-heteroduplex complex, where there is no repulsive interaction between the strands.

### FRET based study of the strand exchange of 20 nt sequences containing three mismatches

**Figure 5.**
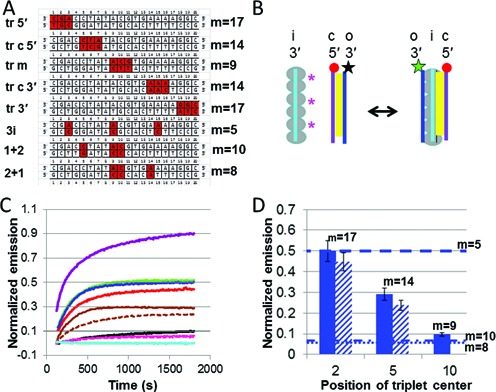
Experiments on 20 nt sequences using the emission of the donor in a FRET pair to study the sensitivity of strand exchange to mismatches. (**A**) Schematic representation of the different sequences used in the strand exchange experiments showing the positions of homologous (white) and non-homologous (red) bases. The largest number of homologous bases in each sequence is indicated to the right. (**B**) Schematic of the strand exchange experiment where the rhodamine fluorophore is shown in red, and fluorescein is black when quenched and green when it can emit. (**C**) Fluorescein emission versus time for fully homologous DNA (purple), tr 3′ (blue), 3i (green), tr 5′ (red), tr c3′ (solid brown), tr c5′ (dashed brown), tr m (black), 1+2 (solid magenta), 2+1 (dashed magenta), pcDNA3 (solid cyan) and het (dashed cyan) (Supplementary Table S2). (**D**) Bar graph summarizing the results shown in panel (C) for tr 3′ (*m* = 17 blue), tr 5′ (*m* = 17 diagonal blue lines), tr c3′ (*m* = 14 blue), tr c5′ (*m* = 14 diagonal blue lines), tr m (*m* = 9), 3i (*m* = 5 dashed line), 1+2 (*m* = 10 dashed line) and 2+1 (*m* = 8 dotted line).

The results presented so far indicate that for some sequences, binding of ssDNA to the presynaptic filament can be much less sensitive to the presence of mismatches than strand exchange. Thus, homology recognition must not simply depend on the mismatch sensitivity of site I base pairing. In order to better understand what does determine homology recognition, we have performed detailed studies of the mismatch-dependent instability of 20 bp interactions containing 17/20 matched bases (Supplementary Table S2). Figure 5A illustrates all the sequences considered in this section. Some of those sequences were also studied in the experiments presented in Figure 1. In Figure 5A, the white boxes represent matched bases and the red boxes represent mismatched bases. All of these sequences with 17 consecutive or non-consecutive matched bases produced readily detectable strand exchange products.

In the strand exchange experiments shown in Figure 1F, the decrease in fluorescence occurs whenever the rhodamine-labeled complementary strand binds to the presynaptic filament in a position where the rhodamine fluorophore can quench the emission due to the fluorescein labeled initiating strand. The close proximity between the complementary and initiating strands does not require strand exchange; therefore, to probe for strand exchange, we conducted a second type of experiment that measures the separation between the outgoing and complementary strands. To monitor the distance between the complementary and outgoing strands, we formed a presynaptic filament using an unlabeled initiating strand (Figure 5B). The unlabeled filament then interacted with dsDNA in which the complementary strand was labeled with rhodamine and the outgoing strand was labeled with fluorescein. Thus, the base pairing between the outgoing and complementary strands quenches the fluorescein emission. Strand exchange separates the complementary strand from the outgoing strand. The increased separation reduces the quenching, so strand exchange results in an increase in the fluorescein emission. Figure 5C shows the fluorescein emission versus time for a number of different sequences. The result for the homologous sequence is shown in purple, and fits to the curves are shown in Supplementary Figure S7.

Importantly, Figure 5C shows that the asymptotic limits of the fluorescence signal for strand exchange reactions containing mismatches are lower than for the homologous case, indicating that they achieve lower equilibrium strand exchange values. This result is consistent with competitive binding assays shown in Supplementary Figure S8. Though all mismatched strand exchange products containing 17/20 matches showed lower fluorescence signals, a large variation can be observed in Figure 5C. Specifically, sequences containing 17 contiguous homologous nucleotides reach high asymptotic fluorescence values. In contrast, when the mismatched triplet is central with 8–9 bp regions of contiguous homology at each side, the equilibrium fluorescence values are low. Surprisingly, systems with three isolated mismatches distributed every 5–6 bp show fluorescence values comparable to the values for mismatched triplets at the ends.

The results shown in Figure 5C are summarized in Figure 5D. The bars show the asymptotic fluorescence values for triplet mismatches. The height of each bar corresponds to the normalized asymptotic value of the fluorescence signal for particular individual sequences with mismatched triplets. The *x* axis label, *k*, refers to the sequences with mismatched triplets and corresponds to the separation between the central base in the mismatched triplet and the nearest dsDNA end. The length of the longest region of contiguous homology is *m* = 19-*k*. Thus, a triplet at the end has *k* = 2 and contains 17 contiguous homologous bases. Figure 5D and Supplementary Figure S9 show that the asymptotic values for the fluorescence are insensitive to the separation between the mismatch and the fluorophore, suggesting that the fluorescence signals dominantly report the formation of complete 20-bp strand exchange products, not local melting near the fluorophores.

For sequences that contained 17/20 matches where the mismatches were not grouped into triplets, the *x* axis has no meaning; however, we wanted to compare the final values for those sequences with those for the mismatched triplets. In order to compare them, Figure 5D includes horizontal lines corresponding to the asymptotic values for three mismatches distributed throughout the sequences.

As indicated by the bar graph in Figure 5D, the results for sequences that are homologous except for one single mismatched triplet suggest that the asymptotic value of the fluorescence increases with m. This is consistent with the result obtained in studies using sequences with m contiguous homologous bases and 20-m contiguous non-homologous bases (28); however, the rule does not apply to other 17-bp matches considered in this work. For example, 3i, the sequence with three isolated mismatches separated by 5–6 contiguous homologous bases has an m value of only 5 but achieved equilibrium fluorescence values comparable to the values for sequences with mismatched triplets at either end (tr 5′ and tr 3′), which have an m value of 17 (Figure 5C, D). Furthermore, the asymptotic fluorescence value for 3i with *m* = 5 is much larger than the values for a central triplet mismatch, tr m, (*m* = 9), as well as the values for sequences 2+1 and 1+2 (*m* = 8 or 10, respectively). Thus, the asymptotic value of the fluorescence is not simply determined by m, the longest region of contiguous homology or by the number of matched bases within the entire 20-nt sequence.

In order to provide another check that in the strand exchange experiments the fluorophores are reporting binding along the 20-nt filament rather than a local interaction occurring near the fluorophore tagged ends, we performed an additional set of experiments for each sequence shown in Figure 5C. In the experiments shown in Figure 5, the fluorophores were attached near the 3′ end of the filament. In contrast, for the results shown in Supplementary Figure S9, the fluorophores were located near the 5′ end of the filament. Consistent with the results shown in Figure 5C, Supplementary Figure S9 indicates that the two sequences with mismatches at the end, tr 5′ and tr 3′ and sequence 3i show similar high fluorescence values, while the 1+2, 2+1 and tr m sequences show much lower values indicating more effective rejection. Both these sets of results are consistent with the results shown in Figure 1F.

### Comparison of the equilibrium fluorescence values obtained from binding to the presynaptic filament, annealing and strand exchange

**Figure 6.**
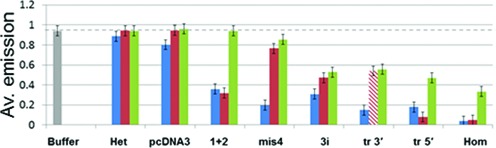
Comparison of the results obtained for ssDNA binding to the presynaptic filament, annealing and strand exchange. The equilibrium values associated with the measured final values of the fluorescence curves were averaged over multiple runs for binding to the presynaptic filament (blue), protein-free ssDNA annealing (red) and strand exchange (green).

Figure 6 shows the measured final values of the fluorescence curves averaged over multiple runs. The results for binding to the presynaptic filament are shown in blue, and the results for protein-free ssDNA annealing are shown in red. Given that the structure of the postsynaptic filament shows the dsDNA is divided into nearly B-form triplets, one might expect similar mismatch rejection in the two systems; however, the mismatch-dependent instability is markedly different. The two systems produce nearly identical results for the homologous and the heterologous sequences, but for pcDNA3, 3i and mis4 sequences, annealing shows significantly better mismatch rejection. This is consistent with the MD results showing that for complete triplets with residues I199 and M164 intercalated in the rises, the postsynaptic filament structure is stable over > 10 ns despite the presence of mismatches, whereas in the absence of intercalation the position of mismatched bases was quite unstable. Interestingly, for the 1+2 sequence and the tr 5′ sequence, binding of complementary ssDNA to the presynaptic filament shows better recognition than in protein-free annealing of ssDNA. For sequence 1+2, the improvement is not statistically significant, but for tr 5′, the improvement is significant.

Though annealing showed poor rejection of the mismatched triplet at the 5′ end, the asymptotic fluorescence values seem to suggest that it shows good rejection of a triplet at the 3′ end. We believe that the apparent good rejection of the mismatched triplet at the 3′ end is an artifact due to the partial opening of the B-form dsDNA near the mismatched end where the fluorophore is located. The opening reduces the FRET signal emitted by partially paired dsDNA; therefore, it represents local melting, not global melting. Since in this case the fluorescence signal does not represent the DNA annealing, the bar representing that result is shown with a diagonal pattern to indicate that the fluorescence may not accurately represent the binding.

The green bars in Figure 6 show the measured final fluorescence values for strand exchange based on experiments like those shown in Figure 1E. For all sequences except 3i, the equilibrium values for strand exchange show better rejection of mismatched base pairings than the equilibrium values for binding to the presynaptic filament. Furthermore, for the sequences 1+2 and tr 5′ strand exchange shows better rejection of mismatched base pairings than annealing.

### FRET based study of the stability of strand exchange products as a function of the number of distributed matches

**Figure 7.**
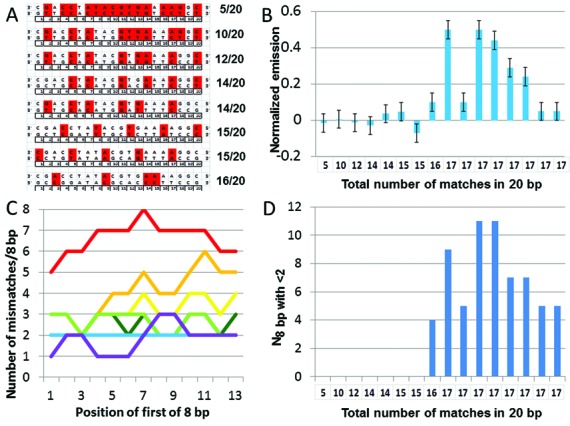
Effectiveness of rejection of mismatched base pairings by strand exchange. (**A**) Schematic representation of 20-nt sequences containing different numbers of distributed matches (white) and mismatches (red). (**B**) Normalized final values for the fluorescence versus time curves as a function of the total number of accidental matches for sequences 5/20, 10/20, 12/20, 14/20 (mis6a and mis6b), 15/20 (mis5 3′and and mis5 5′), mis4 (16), and sequences with 17 matches shown in order for 3i, tr m, tr 3′, tr 5′, tr c3′, tr c5′, 1+2 and 2+1. (**C**) Number of mismatches within the contiguous 8 bp whose first base is located at the position corresponding to the *x* axis value for the sequences shown in panel (A): 5/20 (red), 10/20 (orange), 12/20 (yellow), first 14/20 (light green), second 14/20 (dark green), both 15/20 overlap and are shown in blue, and 16/20 (purple). (**D**) Number of 8-bp regions containing less than two mismatches for the same sequences in panel (B).

In the previous section we dominantly considered interactions that produce detectable strand exchange products. Though studies of sequences with 17/20 matched bases provide useful information about homology recognition, interactions between sequences with 17/20 matched bases rarely occur *in vivo. In vivo*, the most probable interaction would involve 5/20 matched bases randomly distributed along the full 20-nt length. Thus, in order to consider the effectiveness of homology discrimination *in vivo*, we performed strand exchange experiments like those shown in Figure 5, using 20-nt sequences containing different numbers of distributed matches, as illustrated in Figure 7A.

Figure 7B shows the normalized final values for the fluorescence versus time curves as a function of the total number of accidental matches for the sequences illustrated in Figures 5A and 7A. The results show that for sequences containing 14 or fewer matches, no strand exchange product was detected. For sequences containing 15 non-contiguous matches, product formation was barely detectable. In contrast, results shown in Figure 5 indicate that sequences with 17 contiguous homologous bases create strand exchange products that are quite stable.

Previous work had suggested that homology recognition might involve an extremely stringent initial test of ∼8 contiguous base pairs, followed by less stringent testing (29). Such an initial test could explain why the results shown in Figures 5–7 do not simply depend on the total number of matches within 20 nucleotides, since the initial test would only consider a sub-region within the 20 nt sequence. In this study, we tested this proposal using a very simplistic model in which homology recognition involves an initial test of *M* contiguous bp. In the model, the initial test rejects sequences that contain *j* or more mismatches per *M* base pair group. For *j* = 1, only perfectly matched contiguous M base pair groups pass the initial test, whereas for *j* = 2, the system passes contiguous *M* base pair groups that contain either 0 or 1 mismatch. A 20-nt sequence contains (21 − *M*) distinct contiguous *M* base pair groups.

To evaluate the results of the initial tests, we calculated *N*_Mbp with < *j* <*j*_. For two sequences of equal length aligned in registration, *N*_M bp with <*j*_ is the number of distinct groups of *M* contiguous base pairs that contain less than *j* mismatches. If there is no group of *M* contiguous bp that includes less than *j* mismatches, then *N*_M bp with <*j*_ = 0. The model predicts that such an interaction between two 20-nt sequences should not produce detectable strand exchange products, even if the total number of matches per 20 bp is large. In this very simplistic model, the rejection is assumed to be insensitive to the positions of the mismatches in the group. It is also assumed to be insensitive to the nature of the mismatch and the overall composition of the 20-nt sequence. Of course a more realistic model would capture all of these features that we ignored; however, this very simplistic model gives surprisingly good results, as we discuss in detail below.

To provide quantitative information, we compared the measured final fluorescence values with *N*_M bp with <j_ for M values from 4 to 20 and *j* values from 1 to 3, as shown in Supplementary Figures S10 and S11. We note that the experimental results are in poor agreement with any initial test that does not accept any mismatches because the rejection would be too stringent. Each separate curve in Figure 7C corresponds to a particular 20 nt sequence. In Figure 7C, the *x* axis values correspond to the position of first base in each contiguous 8 group within a particular 20 nt sequence. Thus, the *x* axis values range from 1 to 13 since within a 20 nt sequence there are 13 possible positions for the first base in a group of 8 contiguous bp. In Figure 7C, the *y* axis values correspond to the number of mismatches within the contiguous 8 bp whose first base is located at the position corresponding to the *x* axis value. Except for the mis4 sequence with 16/20 matched bases (purple curve), none of the sequences include a contiguous 8 bp group with fewer than two mismatches; therefore, for all of the sequences except mis4, *N*_8 bp with <2_ = 0. In contrast, *N*_8 bp with <2_ = 4 for sequence mis4 since within mis4 there are four groups of 8 contiguous bp that contain less than two mismatches. As Figure 7C shows, the first bases of those four 8 bp groups occur at positions 1, 4, 5 and 6.

The *x* axis in Figure 7B is the same as the *x* axis in Figure 7D; therefore, each *x* position corresponds to a particular sequence. In Figure 7B the bar heights correspond to the final measured fluorescence values versus the total number of matches within each 20-nt sequence, whereas in Figure 7D the height of each bar represents *N*_8 bp with <2_ for the same sequences. Comparison of Figure 7B and D suggests that sequences that form readily detected strand exchange products all contain at least one 8-bp region with less than two mismatches, whereas sequences that do not contain any such regions rarely form stable 20 bp strand exchange products.

Comparison of Figure 7B and D suggest that the model not only captures the rejection of sequences that do not produce detectable fluorescence, but it is also in reasonable agreement with the final fluorescence values observed for sequences that do produce detectable fluorescence. Such a correlation could occur if the probability that an interaction would lead to detectable fluorescence is proportional to the number of possible binding positions that would pass the initial test.

In order to evaluate the relationship between the final value of the fluorescence and *N*_M bp with <j_, we calculated the correlation between the values of *N*_8 bp with <2_ shown in Figure 7D and the final fluorescence values for the sequences shown in Figure 7B. We also calculated the mean square deviation of the difference of the normalized values of the results shown in Figure 7B and D. We made similar calculations for all *M* values between 4 and 14 and all *j* values between 1 and 3. The results of those calculations are shown in Supplementary Figure S12. As discussed above, *j* = 1 values give very poor fits for any *M*. For *j* = 2, the best fit occurs for *M* values between 6 and 10. The results also show that, for *M* < 12, an initial test with *j* = 3 would pass too many interactions; however, *j* = 3 shows correlations that maximize for *M* values between 12 and 16. We believe that the peak corresponds to the period doubling of the *j* = 2 *M* = 8 peak since the *j* = 3 *M* = 12–16 accepts two mismatches in 16 bp (2/14 = 1/7) which is similar to accepting one mismatch in 8 bp.

## DISCUSSION

Previous work had proposed that since the complementary strand backbone has little favorable interaction with the presynaptic filament, the binding of the complementary strand to the presynaptic filament must be governed by the Watson–Crick pairing of the complementary strand bases with the initiating strand bases (12). If this were true, RecA mediated homology recognition could be determined by the sensitivity of that base pairing to the presence of mismatches. Our results show that the binding of the complementary strand to the presynaptic filament is stable even in the presence of mismatches, and that for some sequences the equilibrium binding of ssDNA to the presynaptic filament is much less stringent than the equilibrium binding resulting from strand exchange. Thus, the results show that strand exchange is not governed by the equilibrium binding between the complementary and initiating strands in the heteroduplex.

In what follows we will consider two major questions: (i) What are possible origins of the stable mismatch insensitive binding of the complementary strand in the postsynaptic filament? (ii) How can RecA mediated homologous base pairing *in vivo* rapidly form stable homologous products that may incorporate some mismatches?

### Possible origins of the stability and mismatch insensitivity of the RecA-heteroduplex complex

This work shows that, in the RecA-heteroduplex complex, base pairing is not stressed by interstrand backbone repulsion. Thus, one might expect base pairing in the RecA-heteroduplex complex to be more stable and mismatch insensitive than in B-form dsDNA. We propose that the drastic reduction in backbone repulsion is not the only factor that makes the binding of the complementary strand stable. It is notable that if naked dsDNA is forced to extend to 1.5x the B-form dsDNA length, and the structure has a three nucleotide periodicity, then the minimum energy structure consists of nearly B-form triplets separated by large rises (30,31). Dividing the extended naked dsDNA into stacked triplets separated by rises provides more stability than uniform extension because a large rise that allows the stacking to be preserved within triplets is more favorable than disrupting the stacking between every base (30,31). In the nucleoprotein filament intercalation of residues I199 and M164 at the position of the rises (12) provides a hydrophobic environment that reduces the energetic cost of unstacking. The presence of intercalators may also promote or support the extension of the dsDNA since, even in the absence of protein binding, intercalators can make extended dsDNA more free energetically favorable than B-form dsDNA (32). Importantly, the energy gained by stacking nucleotides in naked ssDNA is comparable to the free energy gained by annealing complementary ssDNAs (33), and stacking makes a large contribution to the stability of dsDNA (34). This important contribution due to stacking is consistent with the results shown in Figure 3 which indicate that the mismatched base pairings only remain stable if they are flanked by intercalating protein residues that help stabilize the intrastrand stacking.

In sum, our studies of the RecA-heteroduplex complex structure suggest that the increased stability and mismatch insensitivity result from at least three effects: (i) A decrease in interstrand repulsion. (ii) An electrostatic barrier that pushes the complementary strand backbone toward the initiating strand (Supplementary Figure S6). (iii) An increase in the stability of the intrastrand stacking due to the intercalation of protein residues.

### The initial 8 bp test

As discussed above, mismatch rejection during strand exchange does not simply depend on the number of matched bases in the 20-nt sequence, or on the maximum number of contiguous base pairs; however, the correlation data suggest that the rejection of mismatched base pairings is consistent with an initial test of ∼8 contiguous bp that accepts up to one mismatch within those 8 base pairs. Though the data require that some single mismatches must pass the initial test, the data do not preclude the possibility that the initial test efficiently rejects some single mismatches.

We propose that the rapid initial testing does not take place in structures with dsDNA triplets in the post-synaptic conformation, but rather in transition states like that shown in Supplementary Figure S13, which may not be characterized by the stable mismatch insensitive binding in the heteroduplex. Since the sparsity of accidental matches allows more than 96% of all base pairings to be rejected by an initial 8-bp test that accepts one mismatch, rapid homology testing is consistent with the observed properties of the heteroduplex. Finally, we speculate that the large increase in stability that is observed when strand exchange products extend to 8 bp is the result of a transition to a structure where 8 contiguous bp occupy the position observed in the heteroduplex crystal structure.

### Previous multiple stage recognition model

Previous experimental (28,35) and theoretical (13,14,36) work has suggested that a major structural transition occurs when the length of the strand exchange product reaches 8 bp as discussed in detail in Supplementary Data. In particular, experiments show that the binding of product with length higher than 8 bp becomes much more stable (28) and the rate at which strand exchange progresses becomes much slower (37). Motivated by those known experimental results, previous theoretical work has shown that the sparsity of accidental matches between two randomly chosen regions of a bacterial genome could allow rapid and accurate homology searching in a multiple stage kinetic recognition model (13,14). The multiple stage kinetic model requires the following conditions that are consistent with previous experimental results (28,37) : 1. The search must include a rapid initial homology test of ∼8 bp. 2. This rapid step is followed by a slower step that iteratively tests the homology of successive base pair triplets. Theoretical work suggested that the initial test need not be terribly stringent, and that the free energy penalty for a mismatched triplet in the slower step could be less than the thermal energy (13). All of these conditions are consistent with the results shown in this work.

### Rejection after passing the initial test

Figure 5 shows that sequences containing mismatches exhibit lower equilibrium binding values than the homologous sequence, and competitive binding assays show that binding also favors the homologous sequences. Although some sequences may be rejected after passing the initial test and before forming a 20-bp strand exchange product, some sequences like 3i, tr 5′ and tr 3′ frequently form 20-bp strand exchange products. Thus, interactions with a high degree of homology may be resolved after the heteroduplex product extends to 20 bp.

Importantly, *in vivo*, strand exchange does not become irreversible until ATP hydrolysis unbinds the heteroduplex from the filament after the strand exchange product has extended to approximately 80 bp (23). Previous theoretical work (13) has shown that if the strand exchange of a matched base is slightly favorable and the strand exchange of a mismatched base is slightly unfavorable, then the sparsity of accidental matches in a genome would allow non-homologous interactions to be rejected by strand exchange reversal before forming irreversible products. Importantly, previous work (38) and the results shown in Figure 5 indicate that strand exchange can progress through short mismatched regions even without ATP hydrolysis; however, the progression becomes less probable as the length of the mismatch increases. Thus, Figure 5 suggests that the mismatch insensitive base pairing in the heteroduplex combined with the sparsity of accidental sequence matches in bacterial genomes can promote efficient unbinding of non-homologous sequences, while allowing homologous sequences containing some mismatches to form stable strand exchange products. Of course, *in vivo*, additional factors may promote rejection of non-homologous sequences that are accepted by RecA mediated recognition. Moreover, recombination between divergent sequences can be effectively inhibited by the DNA mismatch repair machinery involving mismatch-directed dissociation of recombination intermediates (39–43).

For repair and recombination to be rapid and accurate, it is not only important that non-homologous base pairings be rejected, it is also vital that homologous base pairings efficiently form stable strand exchange products. *In vivo*, a homologous sequence that created a 20-bp strand exchange product would continue to extend the product at a rate of ∼6 bp/s until ATP hydrolysis drives irreversible unbinding of the dsDNA from the filament after the strand exchange product extends to ∼80 bp (23).

## CONCLUSION

In sum, if the two step kinetic recognition model that we propose is correct the vast majority of mismatched interactions can be very rapidly rejected by an initial test that considers up to 8 bp that can accept up to one mismatch; however, a combination of the probability distribution governing the number of accidental matches present in interactions between two regions of a bacterial genome and the poor stringency shown in the heteroduplex may allow strand exchange to pass through short mismatched regions within homologous interactions while non-homologous interactions are stringently rejected even if they contain large regions of accidental homology. This combination of stringent rejection of non-homologous interactions and acceptance of short mismatched regions within homologous interactions is highly desirable in living systems. The results and analysis in this work are consistent with this recognition model, as is previous work in RecA (14,28,29,37) and new experimental results for Rad51-mediated strand exchange (35).

## SUPPLEMENTARY DATA

Supplementary Data are available at NAR Online.

SUPPLEMENTARY DATA
