# Supplementary material for: The poor homology stringency in the heteroduplex allows strand exchange to incorporate desirable mismatches without sacrificing recognition in vivo
Source: Nucleic Acids Res. 2015 Jun 18;43(13):6473–85. doi: 10.1093/nar/gkv610 (PMC4513875; doi:10.1093/nar/gkv610)
Supplement: SUPPLEMENTARY DATA [file supp_43_13_6473__index.html]

The poor homology stringency in the heteroduplex allows strand exchange to incorporate desirable mismatches without sacrificing recognition in vivo — SUPPLEMENTARY DATA 

# The poor homology stringency in the heteroduplex allows strand exchange to incorporate desirable mismatches without sacrificing recognition *in vivo*

## SUPPLEMENTARY DATA

- SUPPLEMENTARY DATA
