# Supplementary material for: The poor homology stringency in the heteroduplex allows strand exchange to incorporate desirable mismatches without sacrificing recognition in vivo
Source: Nucleic Acids Res. 2015 Jun 18;43(13):6473–85. doi: 10.1093/nar/gkv610 (PMC4513875; doi:10.1093/nar/gkv610)
Supplement: SUPPLEMENTARY DATA [file supp_gkv610_nar-00836-f-2015-File009.pdf]

SUPPLEMENTARY DATA

The poor homology stringency in the heteroduplex allows strand exchange to incorporate desirable mismatches without sacrificing recognition *in vivo*

Claudia Danilowicz[1]*, Darren Yang[2]*, Craig Kelley[1], Chantal Prévost[3], Mara Prentiss[1#]
[1]Department of Physics, Harvard University, Cambridge, MA 02138, USA.[2]School of Engineering and Applied Sciences, Harvard University, Cambridge, MA 02138, USA.
[3] Laboratoire de Biochimie Théorique, CNRS UPR 9080, Univ. Paris Diderot, Sorbonne Paris Cité, IBPC, Paris, France.

[#] To whom correspondence should be addressed.

*These authors contributed equally to this work.

SUPPLEMENTARY MATERIALS AND METHODS

**Sequences of 20-mer oligonucleotides used in FRET experiments**<br>ssDNA molecules used for site, ssDNA-ssDNA annealing, and strand exchange experiments shown in Figure 1B, D, and F.	
5´ (Rho) CGG AAA AGT GCA TAT CCA GC 3´	Rho c
5´ GCT GGA TAT GCA CTT TTC CG Flu 3´	hom
5´ TGA CCT GTC ATC TCA CCT TC Flu 3´	het
5´ GCC GGA TAC GCA CCC TTC CG Flu 3´	mis4
5´ GTT CCA CTC TAC TGT CCT CT Flu 3´	pcDNA3 mis15
5´ GCC GGA TAC GCA CTC TTC CG Flu 3´	3i
5´ GCT TGA TAC CCA CTT TTC CG Flu 3´	1+2
ssDNA molecules annealed to form dsDNA that was used for strand exchange in Figure 1 F	
5´Rho CGG AAA AGT GCA TAT CCA GC 3´ **c**	
5´ GCT GGA TAT GCA CTT TTC CG 3´ **o**	

Table S1: Sequences of oligonucleotides used for experiments shown in Figure 1. Mismatched bases highlighted in yellow.

Homologous<br>5´ GCT GGA TAT GCA CTT TTC CG 3´ hom
Heterolog 15 mismatches and 5 random matches pcDNA3 (5/20)<br>5´ GTT CCA CTC TAC TGT CCT CT 3´ mis15
Heterolog 10 mismatches and 10 random matches (10/20)<br>5´ GTT GCA CAT GGT TGT TCT CT 3´ mis10
Heterolog 8 mismatches and 12 random matches (12/20)<br>5´ GTT GCA CAT GGA TGT TCC CT 3´ mis8<br>Heterolog 6 mismatches and 14 random matches (14/20)<br>5´ GCT GCA CAT GGA CGT TCC CT 3´ mis6<br>5´ GTT GCA CAT GGA TTT TCC CG 3´ mis6b<br>Heterolog 5 mismatches and 15 random matches (15/20)<br>5´ GCT CGA TTT GCT CTT ATC CC 3´ mis5 5´end<br>5´ CCT GTA TAA GCA GTT TAC CG 3´ mis5 3´end<br>Heterolog 4 mismatches and 16 random matches (16/20)<br>5´ GCC GGA TAC GCA CCC TTC CG 3´ mis4
3 mismatches distributed internally (3i)<br>5´ GCC GGA TAC GCA CTC TTC CG 3´<br>5´ CGG AAG AGT GCG TAT CCG GC 3´ comp3i
3 mismatches at 5´<br>5´ TGC GGA TAT GCA CTT TTC CG 3´ tr 5´
3 mismatches at 3´<br>5´ GCT GGA TAT GCA CTT TTA TC 3´ tr 3´
3 mismatches in the middle<br>5´ GCT GGA TAT CTT CTT TTC CG 3´ tr m
3 mismatches close to 5´<br>5´ GCT GTC GAT GCA CTT TTC CG 3´ tr c5´
3 mismatches close to 3´<br>5´ GCT GGA TAT GCA CAA CTC CG 3´ tr c3´
1 mismatch plus 2 in the middle<br>5´ GCT TGA TAC CCA CTT TTC CG 3´ 1+2
2 mismatches in the middle plus 1<br>5´ GCT GGA TAC CCA CAT TTC CG 3´ 2+1
2 mismatches in the middle<br>5´ GCT GGA TAT CGA CTT TTC CG 3´ d m

Table S2: Sequences of oligonucleotides with mismatches used in FRET experiments shown in Figs. 5, 6, and Supplementary Figures S8 and S9. Mismatched bases highlighted in yellow.

Molecular Dynamics Protocol

We performed our molecular dynamics simulations using the NAMD 2.9 package (1) with the CHARMM 27 force field (version 31) (2,3). The complex was fully solvated using TIP3P water model and was subject to periodic boundary condition. Physiological concentration of Na^+ and Cl^- ions of 0.15 mol/L were added to each simulation to maintain electroneutrality. The resulting dimension and total number of atoms of the system were, respectively, 96 Å x 112 Å x 162 Å and 179,797 for the biggest simulated system. We used 2-fs time step, and the hydrogen atom bond length was constrained using the SHAKE method. We utilized particle-mesh Ewald method for long-range electrostatics calculations, and van der Waals interactions were smoothly switched off at 10–12 Å by a force-switching function. Langevin dynamics scheme was applied to each simulation to maintain the temperature. Nosé-Hoover-Langevin piston was used for pressure control. We simulated three different systems. One structure is a model of the RecA/ssDNA/dsDNA complex resulting from the work described in Yang, D., Boyer, B., Prévost, C. & Prentiss, M., Using simulations to determine properties of transition states governing initial rapid RecA mediated homology recognition, unpublished. The other two structures correspond to the crystal structure of RecA-bound dsDNA (PDB code 3CMX (4) featuring a double-stranded DNA of sequence $(dT)_{13}.(dA)_{11}$ bound to the site I of a pentameric RecA filament and the same system where the adenines of the complementary strand have been replaced by guanines (mismatched DNA). In preparation for the production simulations, each system was first energy-minimized using 5,000 conjugate gradient energy minimization steps followed by slow heating from 30 K to 300 K over 500 ps. During the energy minimization and heating processes, the α-carbon and phosphate atoms of the protein and nucleotide, respectively, were restrained using a soft harmonic potential with a spring constant of 0.5 $kcal.mol^{-1}.A^{-2}$. The system was further allowed to equilibrate for 5 ns in the NPT ensemble with pressure of 1 bar and temperature of 300 K. During the equilibration process only the α-carbon atoms of the terminal RecA monomer and phosphate atoms of the three nucleic acid strands were restrained with a harmonic potential that had a slowly decreasing spring constant from 0.5 to 0.05 $kcal.mol^{-1}.A^{-2}$. In our production simulations, the system was maintained under NPT ensemble conditions and harmonic restraints (0.05 $kcal.mol^{-1}.A^{-2}$) only applied to the α-carbons of terminal RecA monomers. All three systems were submitted to 10 ns of conventional molecular dynamics

simulation. In the case of the RecA/ssDNA/dsDNA complex, this was followed by a 10 ns run where we employed the accelerated molecular dynamics simulation protocol (5) to enhance the conformational space sampling. The threshold energy and boost potential parameters were calculated as suggested by Markwick and McCammon (6).

B-form electrostatic calculations

The calculation was done by using the APBS package (7,8). Hydrogen atoms were added to the crystal structures using PDB 2PQR (9) and charges and radii were assigned according to the CHARMM force field parameters (10). The calculation was performed at a temperature of 300 K, solute and solvent dielectric constants of 4 and 80, respectively, and ion concentration and exclusion radius of 0.2 M and 2.0 Å, respectively. APBS output including structures with 3D surface potentials were visualized using VMD (11)**.**

SUPPLEMENTARY DISCUSSION

Relationship between results shown in Figure 4 and previous theoretical work

The MD simulations shown in Figure 4 indicate that the position of complementary strand bases in dsDNA bound to site I is quite stable even in the absence of base pairing as long as the base triplets are flanked by intercalating protein residues. In contrast, mismatched complementary strand bases rapidly shift their orientation if they are not flanked by intercalating residues. Though flanking residues are required to stabilize the position of mismatched complementary strand bases, matched complementary bases can retain their orientation in the absence of flanking intercalating residues. Thus, Figure 4 suggest that either flanking intercalated residues or hydrogen bonding is sufficient to stabilize the position of complementary strand bases; however, in the absence of both mismatched bases rapidly reorient. These results are consistent with the suggestion that the major source of instability in naked extended dsDNA is the lack of stacking in the rise between the triplets (12,13).

Simple Numerical Model of Pairing

The pairing of the dsDNA to the filament depends on collisions between filaments and dsDNA. If one assumes that a given dsDNA binds to only one single 20 nt filament, then the binding of the dsDNA to the filaments would be proportional to the product of $\rho_{freefilaments}$,the concentration of free filaments, and $\rho_{freedsDNA,}$ the concentration of free dsDNA. Similarly, if the unbinding of dsDNA from the filaments were governed by a single off-rate, k_{off}, then the time derivative of the concentration of bound dsDNA would be given by:

$$d\rho_{boundDNA}/dt = \alpha (\rho_{freefilaments}\, \rho_{freedsDNA}) - k_{off}\, \rho_{boundDNA} \qquad \textbf{Eq. 1}$$

where α is a constant. If the concentration of filaments is initially equally to the density of the dsDNA and one assumes that only one dsDNA binds to one filament, then the equation above becomes:

$$d\rho_{boundDNA}/dt = \alpha (\rho_{initialfree} - \rho_{boundDNA})^2 - k_{off}\, \rho_{boundDNA} \qquad \textbf{Eq. 2}$$

where $\rho_{initialfree}$ is equal to the initial density both the filaments and the dsDNA

If the system includes a state in which the dsDNA is not free in solution, but is also not available for strand exchange then the equations become more complex. In the presence of a hidden state that does not perform strand exchange, equation 2 extends to two equations:

$$d\rho_{boundDNA}/dt = \alpha (\rho_{initialfree} - \rho_{boundDNA} - \rho_{hidden\,DNA})^2 - k_{off_{bound}}\, \rho_{boundDNA} \qquad \textbf{Eq. 3}$$

$$d\rho_{hiddenDNA}/dt = \beta (\rho_{initialfree} - \rho_{boundDNA} - \rho_{hidden\,DNA})^2 - k_{off_{hidden}}\, \rho_{hiddenDNA} \qquad \textbf{Eq. 4}$$

Homology independent binding of the short 20 bp B-form dsDNA to the outer lysines in the C-terminal domains could produce such a hidden state of the dsDNA if that dsDNA binding did not make at least 8 bases available to bind to site II. This issues does not arise *in vivo* where both the dsDNA and the ssDNA-RecA filament are very long, and any binding not at the end of the dsDNA or the filament would position 8 bp where they can do strand exchange. Of course in reality the progression from free dsDNA to the fairly stable strand 20 bp strand exchange product that we observe involves many different transitions each of which may be characterized by

forward and reverse rates. We believe that the time scales for these different transitions vary by many orders of magnitude. Given the enormous complexity of this process, we have not attempted detailed modeling of all possible steps.

In these experiments, we do not detect the binding of the dsDNA to the filament; instead, we detect the formation of stable 20 bp strand exchange products. Thus, the effective α value that appears in these equations represents the effective rate at which free dsDNA and filaments combine to form stable 20 bp strand exchange products. That process may require a large number of intermediate steps, starting with the binding of B-form dsDNA to the filament. In addition, the 6 bp/sec rate of strand exchange implies that ~ 4 seconds would be required to transform free reactants into 20 bp products. The time resolution of these experiments was not sufficient for us to calculate an accurate value of α ($\rho_{freefilaments}$ $\rho_{freedsDNA}$) by measuring the initial fluorescence slopes. Importantly, even an accurate α value would not provide information on the k_{on} rate for the initial pairing of the free reactants since that almost certainly occurs on a timescale that is much faster than the 4 seconds required for strand exchange to form a 20 bp product. Thus, we are unable to experimentally determine k_{on} or α using initial slopes.

We did fit observed fluorescence curves to equation 1 and to equations 4 and 5. The results are shown in Supplementary Figure S7. Best fits to equation 1 provide either good fits at long times or good fits at short times, but not both. In contrast, equations 4 and 5 combined fit all times very well. It is possible that simply having more free parameters available accounts for the improved fit, or it is possible that the system includes a non-fluorescing bound conformation; however, as discussed above, that conformation may be an artifact of the short sequence lengths in these *in vitro* experiments. Thus, we are reluctant to assign great significance to the values obtained from the fits.

We also did fits to a second set of equations that assumes that there is a second state,2, that can only be reached from an initial state, 1, then the time dependence equations for the densities in the two states become

$$d\rho_{bound1}/dt = k_{on1} (N_{total}-N_{bound1}- N_{bound2})(N_{total}-N_{bound1} - N_{bound2}) - k_{off1} N_{bound} \quad \textbf{Eq. 5}$$

$$d\rho_{bound2}/dt = k_{on2} N_{bound1} - k_{off2} N_{bound2} \quad \textbf{Eq. 6}$$

Measurements of strand exchange taken using the system illustrated in Figure 5B are shown in Supplementary Figure S7. The solid lines represent fluorescence vs. time curves for strand exchange of 20-nt systems. The dashed lines show fits of equations 3 and 4 are shown in Supplementary Figure S7A and fits of equations 5 and 6 to the same data are shown in Supplementary Figure S7B. The residuals are from the fits are shown in Supplementary Figures S7C and S7D. For the results shown in figure A the fit parameters are k_{on} = 0.003 x10^6 M^{-1} s^{-1}, k_{off} = 0.0001 s^{-1}, $k_{onhidden}$ = 0.001 s^{-1}, $k_{offhidden}$ = 0.0005 s^{-1}, and the dotted lines correspond to the population in the hidden state that is not detected by fluorescence. For the results shown in Supplementary Figure S7B, dashed dotted and dotted lines correspond to the population in state 1 and state 2, respectively. The corresponding fit parameters are k_{on1} = 0.02 x10^6 M^{-1} s^{-1}, k_{off1} = 0.006 s^{-1}, k_{on2} = 0.002 s^{-1}, k_{off2} = 0.00035 s^{-1}.

Probing strand exchange reversal using dsDNA competition

If near matches are rejected *in vivo* despite forming 20-bp strand exchange products, the near matches must reverse strand exchange even though the binding in the heteroduplex is fairly stable and mismatch insensitive. Thus, to study how homology recognition might proceed *in vivo,* we tested if nearly matched sequences can unbind from the filament after forming 20-bp strand exchange products.

In these tests, we allowed fluorescently labeled dsDNA containing 3 internal mismatches (3i) to interact with ssDNA-RecA filaments (Supplementary Figure S8A). The change in FRET signal shows that the nearly matched dsDNA rapidly formed stable 20-bp strand exchange products (Supplementary Figure S8B). After ~ 30 minutes, the fluorescence neared its asymptotic value. Once that asymptotic value was reached, unlabeled dsDNA homologous to the filament was added. The ratio between nearly matched and homologous dsDNA was 6:1. The arrow in Supplementary Figure S8B indicates the time when the unlabeled homologous dsDNA was added to the solution. Supplementary Figure S8C is a schematic of the interaction after the addition of the unlabeled homologous dsDNA. If the nearly matched strand exchange products did not reverse and unbind, the fluorescence would retain its asymptotic value because the strand exchange product maintains the separation between the FRET pairs. In contrast, if the nearly

matched dsDNA unbinds and forms B-form dsDNA again, the FRET signal would decrease because the fluorophores that were initially separated in the strand exchange product would reanneal forming labeled B-form dsDNA. This result indicates that nearly matched dsDNA that had formed 20-bp strand exchange product reversed strand exchange, as required for such mismatches to be rejected *in vivo.* Furthermore, the result shows that the formation of homologous strand exchange products is more favorable than the formation of nearly matched strand exchange products.

Two control experiments were performed for this competition assay where the filament was mixed initially with homologous labeled dsDNA. In one case, after 30 minutes, unlabeled homologous dsDNA was added. If the reaction reverses, the unlabeled dsDNA would replace the labeled dsDNA forming the heteroduplex product. The unbinding of labeled dsDNA should allow fluorophores to FRET showing a decrease in the fluorescein emission. Supplementary Figure S8B shows that there is a negligible effect upon the addition of unlabeled homologous dsDNA (purple curve) showing that the reaction does not reverse significantly. Similarly, after incubating the filament with labeled homologous dsDNA for 30 minutes, the strand exchange product was challenged with unlabeled dsDNA, prepared by annealing 3i to comp3i, thus containing three internal mismatches when compared to the filament. The black curve shows that there is negligible displacement of labeled dsDNA from the initially formed heteroduplex product.

Evidence from previous work suggesting RecA uses a two stage kinetic recognition system beginning with a homology test of ~ 8 contiguous bp

Seminal experiments measured the stability of strand exchange products as a function of m, the number of contiguous homologous bp in 20-nt sequences. In those studies, the m-base pairs were located at one end of the dsDNA, and the remaining 20-m base pairs were all mismatched. The results showed that sequences with m< 8 did not show significant protection against cutting by restriction enzymes (17). The experiments also showed that the protection increased with m and did not depend on which end contained the contiguous homologous bp. Experiments have also shown that strand exchange is characterized by a very fast initial exchange of 12 ±3 bp, followed by a much slower strand exchange process (14). We speculate that the strand exchange products that protected against cutting included at least 8 contiguous bp

in the heteroduplex, and we will refer to structures with 8 or more bp in the heteroduplex as metastable structures. In contrast, we propose that initial homology tests occur in unstable transition states in which 8 bp occupy positions where strand exchange is possible, but 8 contiguous bp do not yet occupy the heteroduplex.

Importantly, theoretical work (18) suggests that dsDNA binding to site II can rapidly position ~8 contiguous bp where they can do strand exchange; however, theoretical work (18) and single molecule studies (19) indicate that dsDNA tension prevents more dsDNA from being available for strand exchange unless those original ~8 bp pass the homology tests that govern the transition to the metastable structure. Thus, dsDNA tension would create a decision point that allows up to 8 bases to be very rapidly tested for homology, but does not allow more bases to be tested unless the first 8 contiguous bp assume the dsDNA structure characteristic of the heteroduplex.

Detailed simulations of the time evolution of transition states are shown in Yang, D., Boyer, B., Prévost, C. & Prentiss, M., Using simulations to determine properties of transition states governing initial rapid RecA mediated homology recognition, unpublished, and an example structure is shown in Supplementary Figure S13. These simulations suggest the base pairing in transition states is much less stable than the pairing in the heteroduplex. The instability of the base pairing in transition states could allow initial homology recognition to proceed much faster than recognition after the transition to the heteroduplex which results in this work suggest is characterized by stable mismatch insensitive binding of the complementary strand.

Evaluation of the relationship between asymptotic fluorescence values and the number of M base groups containing 0, 1, or 2 mismatches

Figure 7B shows the asymptotic value of the fluorescence for a number of sequences. Similarly, Figure 7D shows $N_{8bp\ with\ <2}$ for the same sequences. Thus, Figure 7D shows the number of 8 bp groups containing one mismatch. Each graph can be described by an array with values $((x_1,y_1), (x_2,y_2)\ldots(x_N,y_N))$ (For each pair of values, the x value is a number corresponding to a particular sequence (e.g. heterologous ssDNA = 1, 20 mer from pcDNA3 = 2, etc). In the array representing the fluorescence results, $((x_1,y_1), (x_2,y_2)\ldots(x_N,y_N))$ the y values correspond to the final measured value of the fluorescence for that sequence. In the array representing the

distribution of mismatches, $((x_1,y'_1), (x_2,y'_2)\ldots(x_N,y'_N))$, the y' the values are $N_{M\ bp\ with<j}$, where M is the size of the group of bases being considered, and j is an integer representing the lowest number of mismatches rejected by the system. Thus $N_{mis/8,<2}$ is the number of unique 8 bp groups that contain fewer than 2 mismatches. Results for M=4 to 16 and j values 1, 2, or 3 are shown in Supplementary Figure S10. To calculate the correlation we took the sum of the product of ith y value in the fluorescence array and the i+offset y' value in the $N_{M\ bp\ with<j}$ array.

$$\text{Total (offset,M,j)} = \sum_i y_i \ y'_{i+offset} \qquad \textbf{Eq. 7}$$

For each M and j value, we plotted Total (offset,M,j) values for each M. If the two arrays are correlated, then then Total (offset,M,j) will be peak at offset=0. If the arrays are uncorrelated, shifting should product no difference. Thus, the results should be insensitive to the offset value and plots of Total (offset,M,j) vs. offset will be relatively flat. If the arrays are well correlated, the unshifted arrays should give the maximum value, and the values should decrease strongly as the offset increases. For each value of M and j we calculated the difference between the maximum and minimum values of Total (offset,M,j). The results are shown in Supplementary Figure S12. For j=1, which corresponds to the case with no mismatches, there is no significant correlation and the individual graphs of Total (offset,M,j) vs. offset do not even peak at zero offset. For j=2, which corresponds to less than 2 mismatches, the Total (offset,M,j) vs. offset do peak at zero offset. There is a significant correlation in the difference between the maximum and minimum values as a function of M. The largest difference between the maximum and minimum values is achieved at M=8. For j=3, there is also significant correlation and the largest difference is achieved at M=12. We believe that the j=3 peak represents the period doubling of the j=2 results since 1/8 mismatches ~ 2/12 mismatches.

We also calculated the mean square deviation between the normalized asymptotic fluorescence values and the normalized values of $N_{M\ bp\ with\ <j}$. For the j=1 case, the variation is not statistically significant. For j=2, the variation is significant and the minimum value of the mean square deviation occurs at M=8-10. For j=3, the minimum value occurs at M=12 to 14.

In sum, the two different methods of evaluating the relationship between the measured asymptotic fluorescence value and $N_{M\ bp\ with\ <j}$ suggest that there is a relationship and that the correlation is best for M~8 and j=2, indicating that the testing group size is approximately 8 and that the initial test can accept up to 1 mismatch. The results do not preclude rejection of some single base pair mismatches.

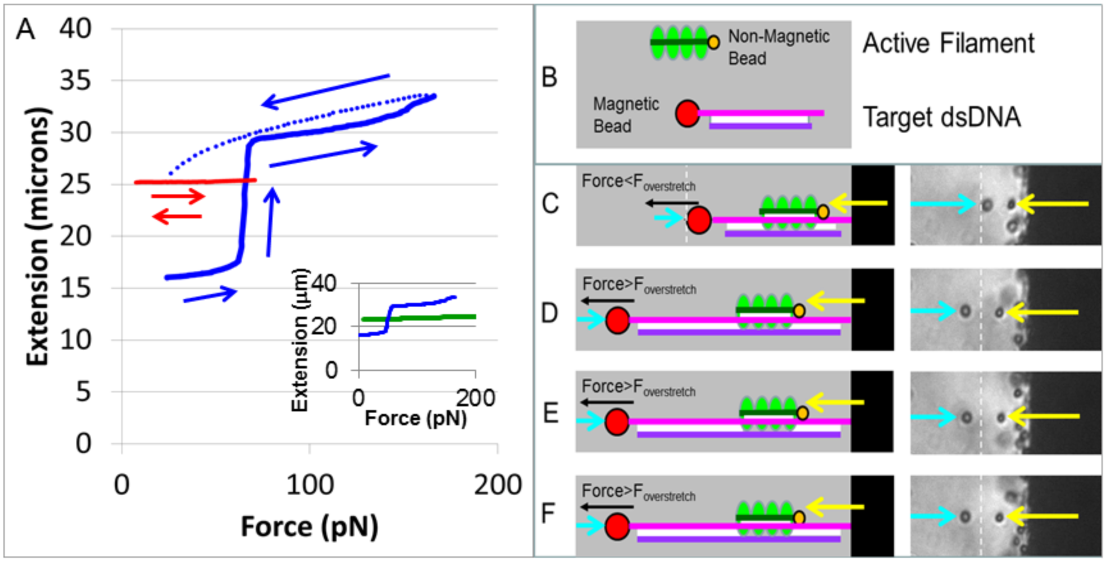

Supplementary Figure S1 Single molecule experiments probing the stability of complementary strand binding in the post-synaptic conformation by pulling on the 3´5´ ends of one strand of the dsDNA. (A). Extension vs. force curves for naked dsDNA (blue) and dsDNA bound to site I (red), where the arrows indicate the direction of time. An overstretching transition that increases extension by ~ 1.7x occurs when the force on naked dsDNA exceeds ~ 65 pN, but no increase is observed in the RecA filament. The naked dsDNA results were already presented in previous work (20). The naked dsDNA completes shearing at ~ 180 pN, so the decreasing force curve corresponds to ssDNA. The RecA bound dsDNA created by binding RecA to dsDNA neither overstretches nor shears (green curve in the inset). **(B)**. Schematic of the experiment to measure the stability of the complementary strand binding in the post-strand exchange conformation. The red and orange spheres correspond to the magnetic and non-magnetic beads. The dark green, magenta, and purple lines correspond to the initiating, complementary, and outgoing strand backbones. The green ellipses indicate the region occupied by the protein. **(C).** Results for a force below the overstretching transition. (**D, E, and F)** show images for a force just above the overstretching transition where the unbound regions of the dsDNA overstretch, but suggest the pulled complementary strand is stably bound to the filament after strand exchange has taken place.

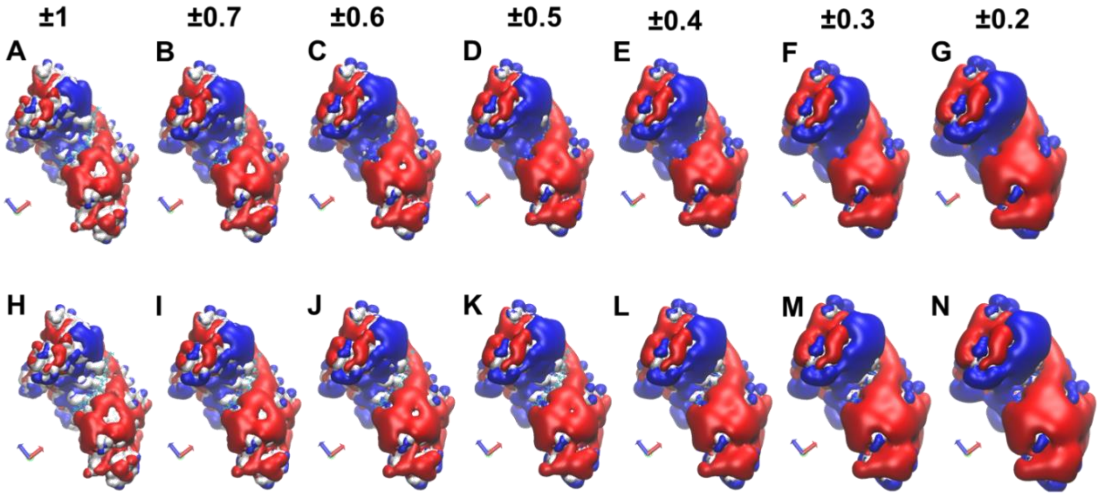

Supplementary Figure S2 Isopotential surfaces of the RecA protein only and active filament. Blue and red represent the positive and negative surfaces, respectively. The initiating and complementary strands are shown in magenta and cyan, respectively. **(A-G)**. Surfaces for the RecA protein only filament with isopotential values ±1, ±0.7, ±0.6, ±0.5, ±0.4, ±0.3, and ±0.2, respectively. **(H-N)** analogous isopotential surfaces for the RecA active filament. As expected, the major difference between the isopotentials for the two filaments is that the region near the initiating strand is more negative in the active filament than in the protein alone. Similarly, the region near the L2 loop and the complementary strand is more positive in the active filament, resulting in a neutral region over the volume that the complementary strand traverses in going between the site II bound conformation and its position in the post-synaptic conformation. This neutrality allows the complementary strand to reposition with minimal potential energy cost.

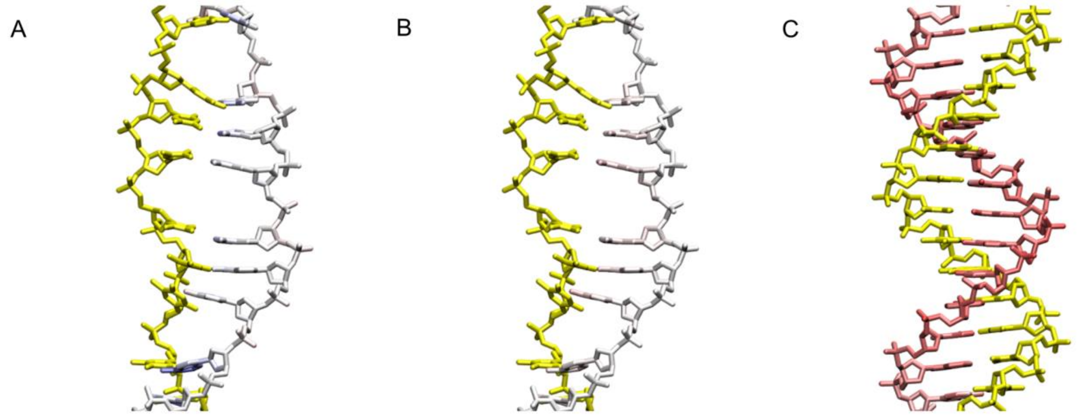

Supplementary Figure S3 Comparison of the electrostatic potentials extended dsDNA and B-form dsDNA. Blue corresponds to +3 kT/e, white is neutral, and red corresponds to -3 kT/e. **(A).** Electrostatic map created by the active RecA/ssDNA (initiating strand) nucleoprotein filament at the surface of the complementary strand bound in site I (stick representation). The protein is not represented and the initiating strand is represented in yellow **(B).** Calculation for the dsDNA conformation shown in (A) in solution in the absence of the protein, indicating that free ions mimic the charge distribution in the active filament. **(C).** Calculation for B-form dsDNA analogous to (B) showing that negative potential at the complementary strand position is much larger in B-form than in the extended structure.

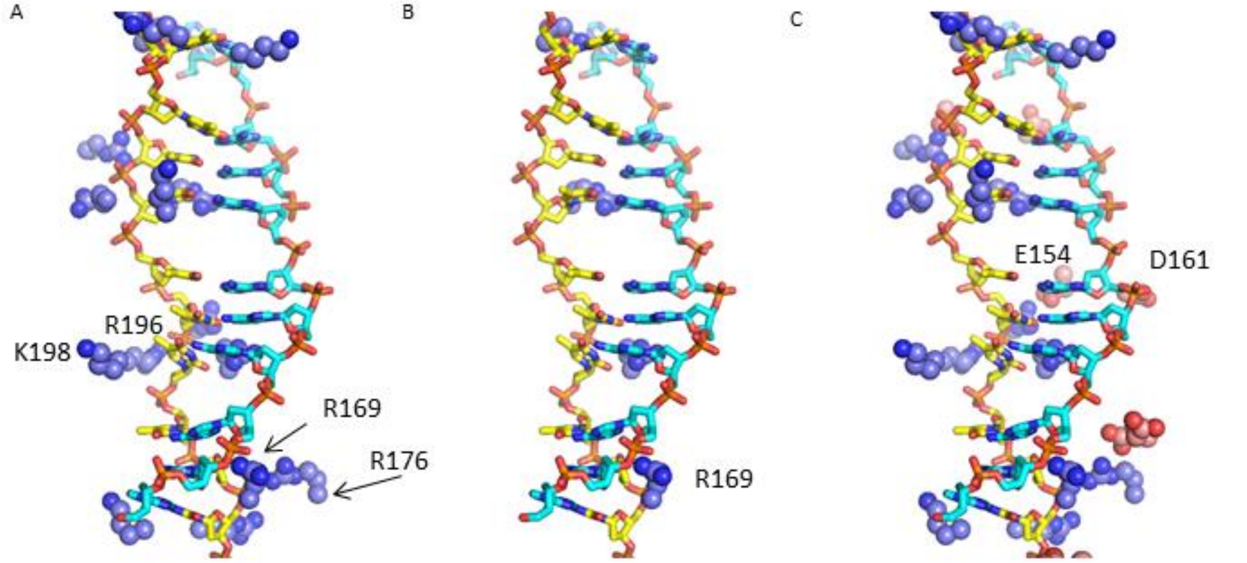

Supplementary Figure S4 Charged residues near the initiating and complementary strands in site I. Positive residues are shown in blue and negative residues are shown in red. **(A)**. Charged residues within 5 Å of the initiating strand which are residues R169, R176, R196, and K198. All of the charged residues within 5 Å of the initiating strand are positive. **(B)**. The only charged residue within 5 Å of the complementary strand is R169. It is positioned between the initiating and complementary strands. **(C)**. All charges within 7 Å of both initiating and complementary strands including two negative charged residues E154 and D161 are shown in red.

A B

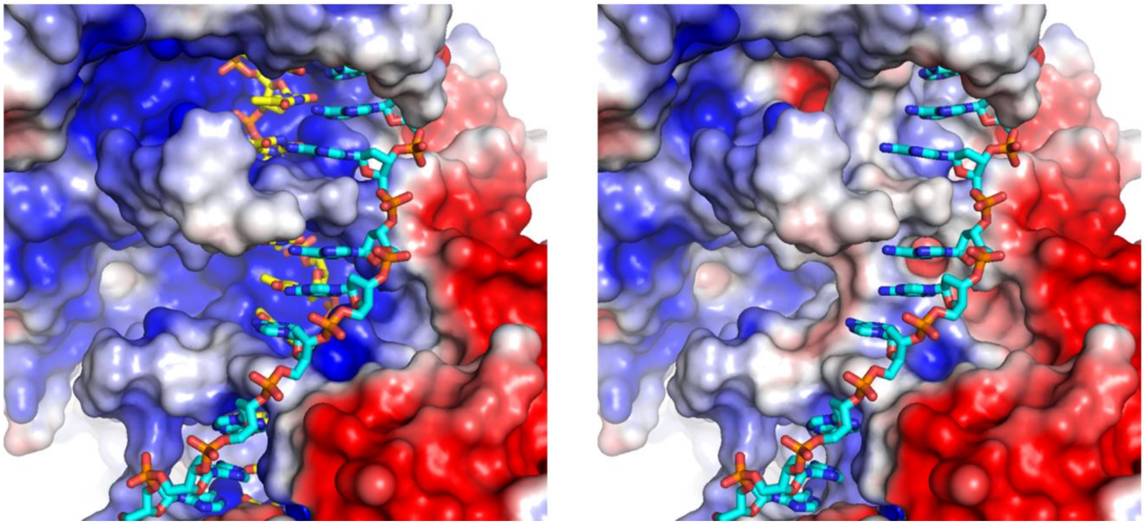

Supplementary Figure S5 Site I Electrostatic potential maps. (A). Potential map of the protein filament at the surface of the protein, where blue corresponds to +3 kT/e, white is neutral, and red corresponds to -2 kT/e. **(B)**. Same as (A) but for the active filament including the effects of charges in the initiating strand. The complementary strand is shown to indicate the position it occupies when dsDNA is bound to site I, which is in a neutral region separating regions with strong positive and negative potentials. The complementary strand coloring represents the atomic content, not the potential.

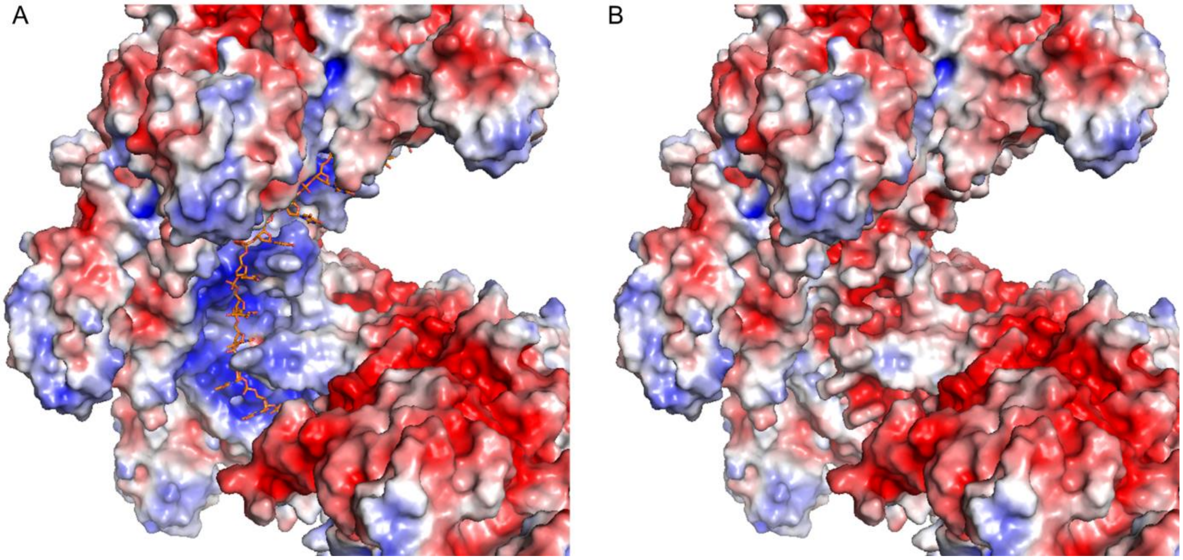

Supplementary Figure S6 Site II Electrostatic potential maps. (A). Potential map of the active filament at the surface of the protein, where blue corresponds to +3 kT/e, white is neutral, and red corresponds to -3 kT/e. The position of the outgoing ssDNA obtained by MD simulations is indicated in orange. **(B)**. Same as (A), but for the active filament with ssDNA bound to site II. As expected, the binding of ssDNA to site II makes the potential near site II almost neutral, whereas it was quite positive in the active filament; however, the potential changes very little in the region near the position of the complementary strand in site I bound dsDNA.

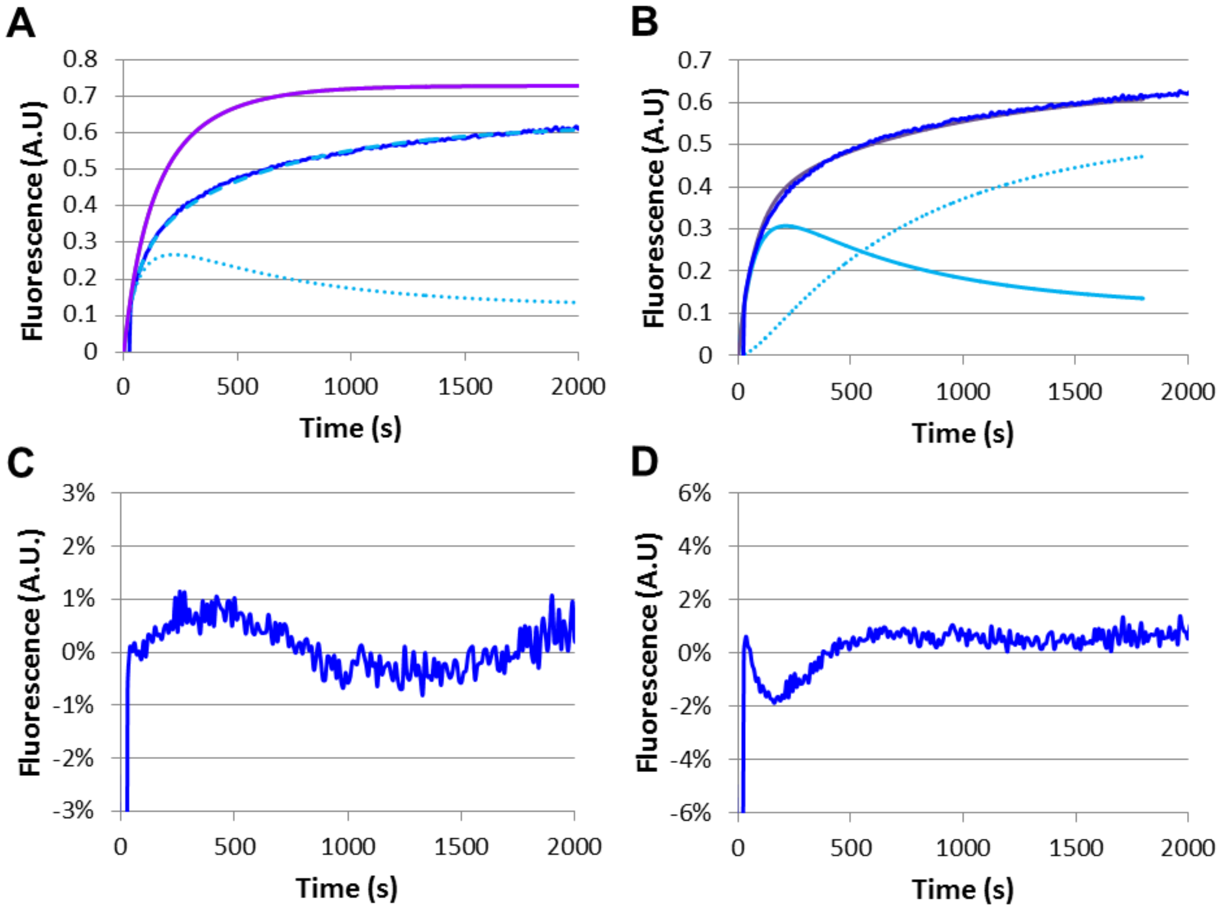

Supplementary Figure S7 Fits to the fluorescence vs. time curves generated by strand exchange in systems containing 20 homologous bases. (A). The solid blue curves correspond to the data. The dashed lines show the fits to equations 3 and 4. The dotted lines show the population in the hidden state that is not detected by fluorescence. The purple line shows the total bound. **(B).** The solid dark blue line is the same data shown in (A). The light blue lines show the fits for equations 5 and 6. The solid and dotted light blue lines show bound populations of the first and second bound states, respectively. The purple shows the total for both states. **(C).** Residuals for the fits shown in (A). **(D).** residuals for fits shown in (B).

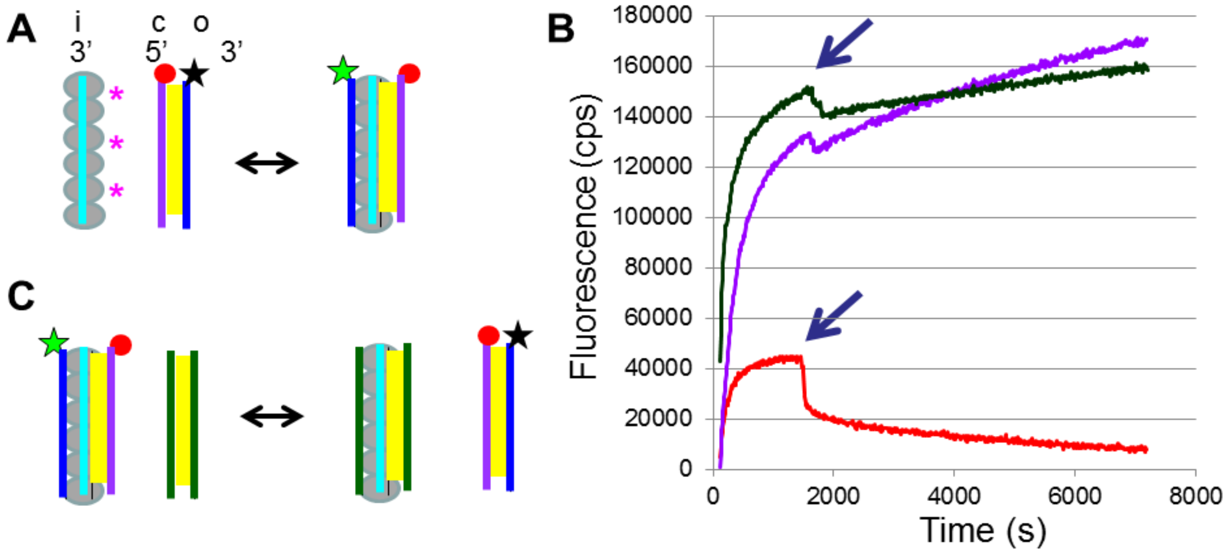

Supplementary Figure S8 Competitive binding assay probing the unbinding of strand exchange products formed by labeled dsDNA. (A). Schematic of the initial interaction between the fluorescently labeled dsDNA and the unlabeled filament. This figure is the same as Figure 5B (main text); however, in this experiment the ratio between dsDNA and the filament is 6:1, whereas in Figure 5C the ratio was 1:1. **(B).** Fluorescence vs. time curves observing the FRET signal due to the addition of labeled dsDNA, prepared by annealing Rho c and hom Flu, to unlabeled filament with 3i ssDNA (red) or hom ssDNA (purple and black curves) (sequences in Supplementary Tables S1 and S2). After the initial reaction has proceeded for about 30 minutes, another unlabeled dsDNA was added at a 1:1 ratio with respect to the filament: dsDNA homologous to 3i filament (red), dsDNA homologous to hom filament (purple), and dsDNA homologous to 3i, so presenting 3 mismatches with respect to hom ssDNA filament (black). The time at which the unlabeled dsDNA was added is indicated by the arrows. **(C).** Schematic of the interaction after the introduction of unlabeled dsDNA whose backbones are indicated by dark green lines.

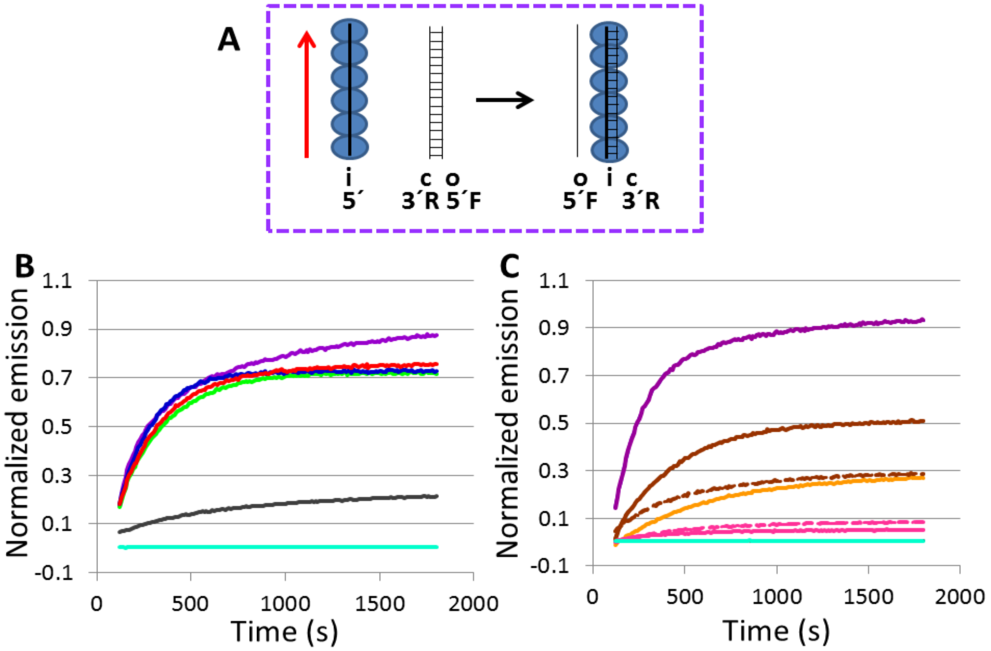

Supplementary Figure S9 Experiments on 20 nt sequences using the emission of the donor in a FRET pair to study the sensitivity of strand exchange to mismatches. (A). Schematic representation of experiments in which the fluorophores were placed on the end of the dsDNA that aligns with the 5′ end of the initiating strand. **(B)**. Results shown are typical curves from single experiments of completely homologous (purple) and heterologous (cyan) sequences as well as sequences containing mismatches at different positions within the 20 nt. The solid blue and red lines represent the results for triplet mismatches at the 3´ (tr 3´) and 5´ (tr 5´) ends, respectively. The gray curve is the result for a mismatched triplet at the center of the sequence (tr m), and the green curve shows the results for 3 isolated mismatches separated by 5-6 bases (3i). **(C)**. Results shown are typical curves from single experiments of completely homologous (purple) and heterologous (cyan) sequences. The solid and dashed brown lines correspond to mismatched triplets positioned 4 bases from the 3´ (tr c3´) and 5´ (tr c5´) ends, respectively. The orange curve corresponds to the sequence with only two mismatches in the middle (d m) whereas the solid and dashed magenta curves correspond to 1+2 and 2+1 sequences, respectively.

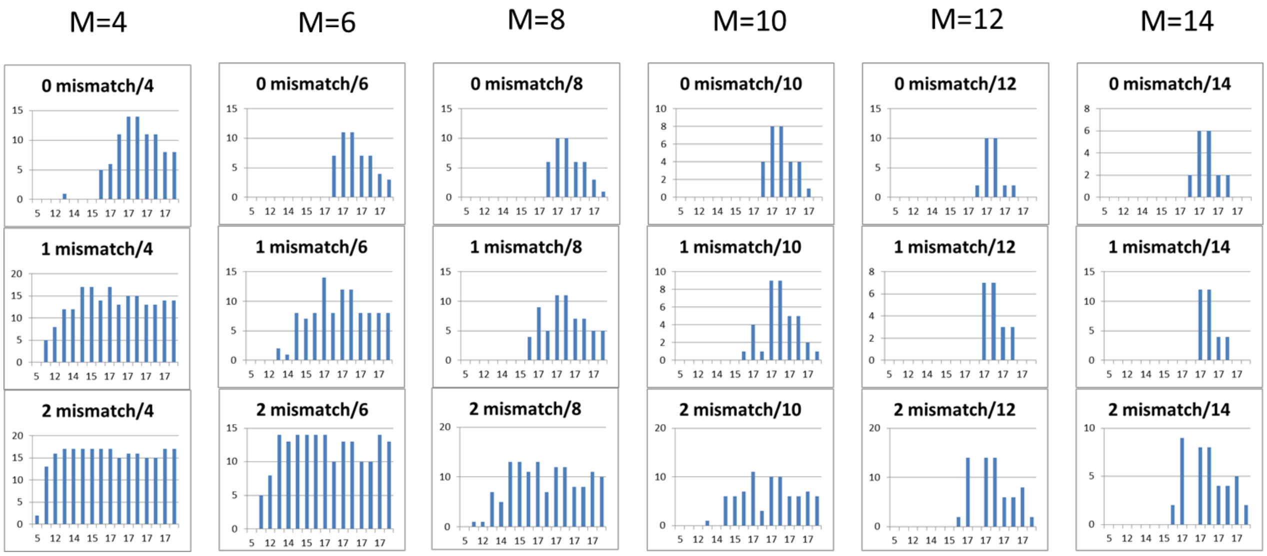

Supplementary Figure S10 $N_{M\ bp\ with\ <j}$ for M values from 4 to 14 (columns) and j values from 1 to 3 (rows). The x axis corresponds to different sequences, where the order of the sequences is 5/20, 10/20, 12/20, 14/20 (mis6a and mis6b), 15/20 (mis5 3´and mis5 5´), mis4 (16), and sequences with 17 matches shown in order for 3i, tr m, tr 3´, tr 5´, tr c3´, tr c5´, 1+2 and 2+1. The x axis labels correspond to the number of matches within the 20-nt comparison.

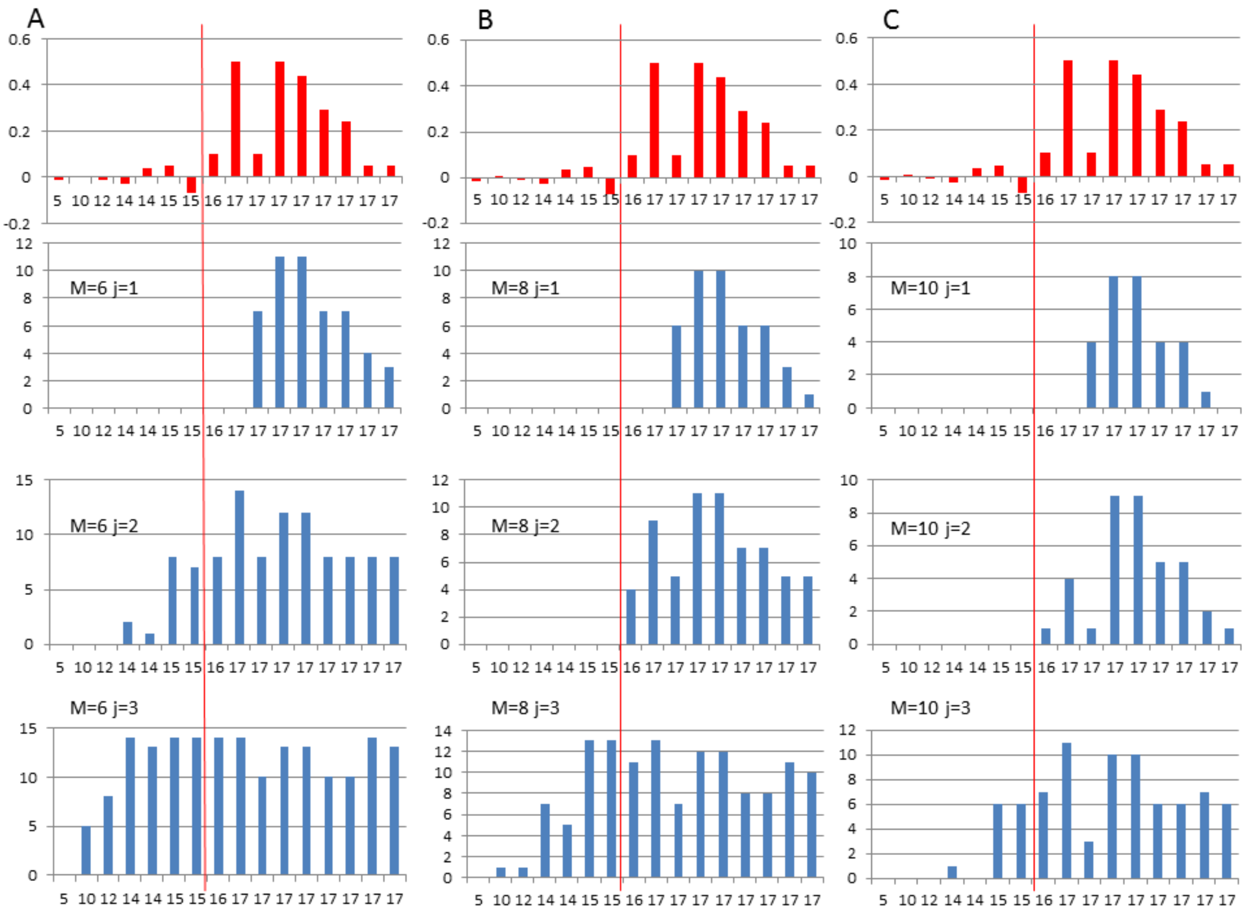

Supplementary Figure S11. **Comparison between asymptotic values of the fluorescence (red graphs) and $N_{M\ bp\ with\ <j}$ for M values from 6 to 10 (columns) and j values from 1 to 3 (rows).** The x axis corresponds to different sequences, where the order of the sequences is 5/20, 10/20, 12/20, 14/20 (mis6a and mis6b), 15/20 (mis5 3´ and mis5 5´), mis4 (16), and sequences with 17 matches shown in order for 3i, tr m, tr 3´, tr 5´, tr c3´, tr c5´, 1+2 and 2+1. The x axis labels correspond to the number of matches within the 20 nt comparison. The vertical red line marks the boundary where the asymptotic fluorescence becomes readily detectable. The M=6 graphs are not consistent with the demarcation, but both the M=8 and M=10 graphs are.

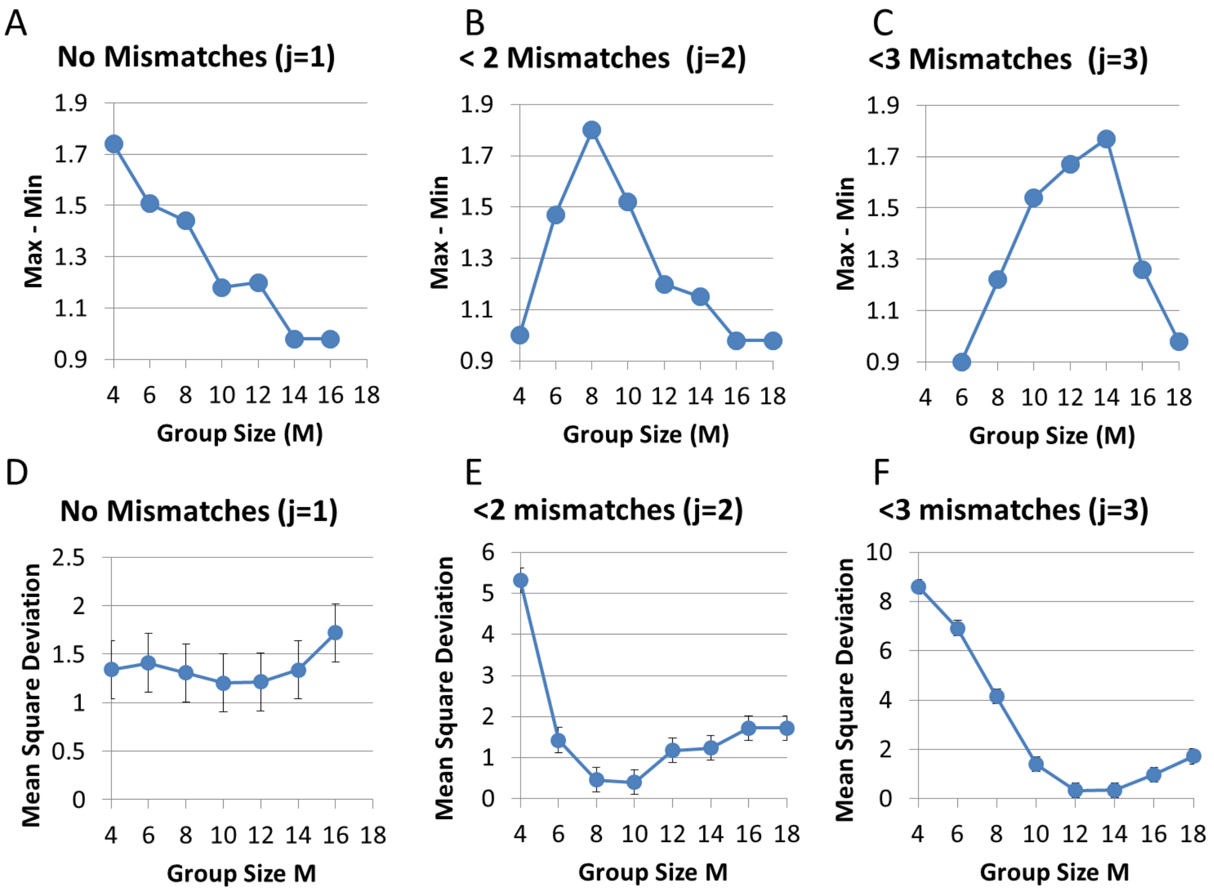

Supplementary Figure S12 Relationship between the asymptotic value of the fluorescence and the number of M bp groups containing 0, 1, or 2 mismatches. (A). Difference between the maximum and minimum values of the correlation function vs. offset for different values of M. The result shown is for 0 mismatches, which corresponds to j=1. It does not achieve a maximum within the interval. **(B).** Same as (A) except for j=2, which corresponds to groups containing no more than 1 mismatch. It peaks at M=8 bp, suggesting that there is a good correlation between the asymptotic value of the fluorescence and the number of M bp groups containing no more than 1 mismatch. **(C).** Same as (B), except for j=3 mismatches. The peak at ~ M=13 may correspond to double the period of the single match result. **(D)**. Square deviation between the two arrays as a function of M for j=1, showing no significant variation with M, thus indicating no correlation. **(E)**. Same as (D) but for j=2. There is a significant minimum at M=8-10. **(F).** Same as € but for j=3. There is a significant minimum at M=12 to 14.

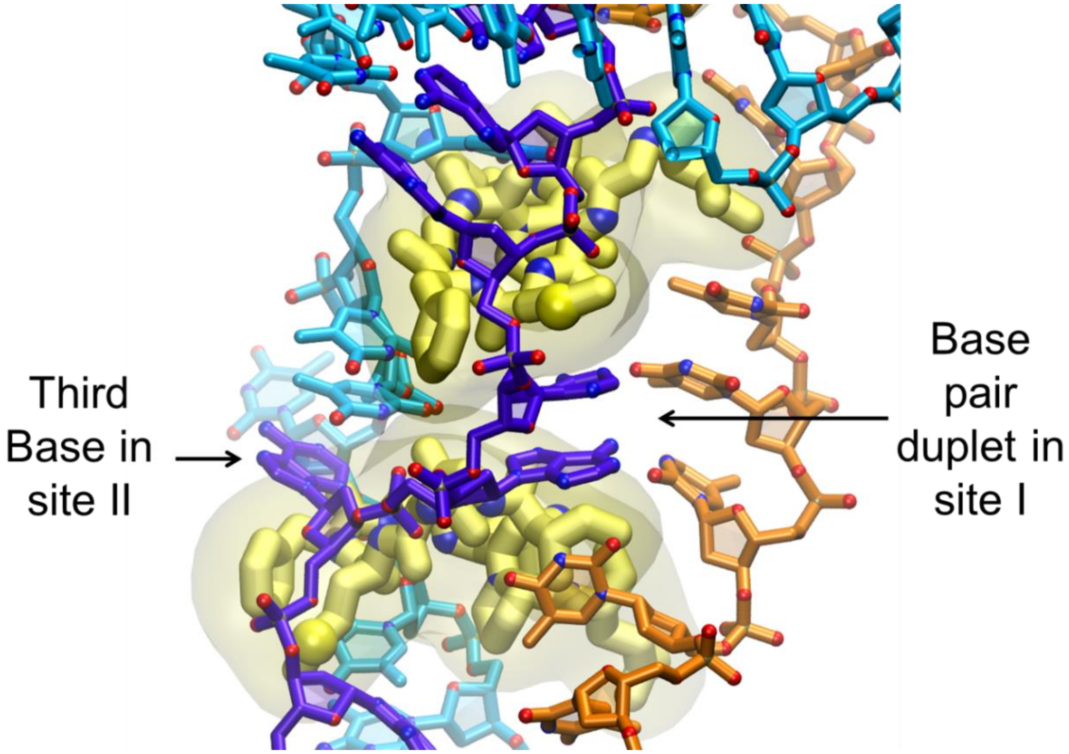

Supplementary Figure S13 Example of a transition state structure obtained after 10 ns MD simulation followed by 4 ns accelerated MD simulation. The simulation details are described in Supplementary Data (Methods - Molecular Dynamics Protocol). The initial structure had dsDNA bound to site II in a conformation with ~ 8 contiguous complementary strand bases in a position where they can attempt strand exchange. The cyan, purple, and orange strands represent the outgoing, complementary, and initiating strands, respectively. L2 loop residues are shown in yellow. In this conformation, a complementary strand base duplet is paired with a base duplet in the initiating strand, where the conformation of the DNA near the duplet is close to the structure of the heteroduplex dsDNA in the postsynaptic filament. In contrast, the third complementary strand base that originally formed a triplet with the two flipped bases remains nearly in its initial site II bound conformation. In this transition state, the Watson-Crick base pairing between two bases of the incoming strand and the two flipped bases of the complementary strand is unstable, as hydrogen bonds keep forming and breaking on the ns scale, as indicated in Yang, D., Boyer, B., Prévost, C. & Prentiss, M., Using simulations to determine properties of transition states governing initial rapid RecA mediated homology recognition, unpublished. The base pairing of the duplet is much more unstable than the pairing of complete triplets that is shown in Figure 4.

Supplementary references

1. Phillips, J.C., Braun, r., Wang, W., Gumbart, J., Taikhorshid, E., Villa, E., Chipot, C., Skeel, R.D., Kalé, L. and Schulten, K. (2005) Scalable molecular dynamics with NAMD. *J. Comp. Chem.,* **26,** 1781-1802.
2. Foloppe, N. and MacKerell Jr., A.D. (2000) All-atom empirical force field for nucleic acids: I. Parameter optimization based on small molecule and condensed phase macromolecular target data. *J. Comp. Chem*., **21**, 86-104.
3. MacKerell Jr., A.D., Bashfor, D., Dunbrack, Jr.R.L., Evanseck, J.D., Field, M.J., Fischer, S., Gao, J., Guo, H., Ha, S. *et al*. (1998) All-atom empirical potential for molecular modeling and dynamics studies of proteins. *J. Phys. Chem. B,* **102**, 3586-3616.
4. Chen, Z., Yang, H. and Pavletich, N.P. (2008) Mechanism of homologous recombination from the RecA-ssDNA/dsDNA structures. *Nature,* **453,** 489-494.
5. Hamelberg, D., Mongan, J. and McCammon, J.A. (2004) Accelerated molecular dynamics: a promising and efficient simulation method for biomolecules. *J. Chem. Phys.*, **120**, 11919-11929.
6. Markwick, P.R. and McCammon, J.A. (2011) Studying functional dynamics in bio-molecules using accelerated molecular dynamics. *Phys.Chem.Chem.Phys.,* **13**, 20053-20065.
7. Unni, S., Huang, Y., Hanson, R.M., Tobias, M., Krishnan, S., Li, W.W., Nielsen, J.E. and Baker, N.A. (2011) Web Servers and Services for Electrostatics Calculations with APBS and PDB2PQR. *J. Comp. Chem***., 32**, 1488-1491.
8. Baker, N. A., Sept, D., Joseph, S., Holst, M.J. and McCammon, J. A. (2001) Electrostatics of nanosystems: Application to microtubules and the ribosome. *Proc. Natl. Acad. Sci.U.S.A.,* **98**, 10037-10041.
9. Dolinsky, T. J. Czodrowski, P., Li, H., Nielsen, J.E., Jensen, J.H., Klebe, G. and Baker, N.A. (2007) PDB2PQR: expanding and upgrading automated preparation of biomolecular structures for molecular simulations. *Nucleic Acids Res.,* **35,** W522-W525.
10. Foloppe, N. and MacKerell Jr., A.D. (2000) All-atom empirical force field for nucleic acids: I. Parameter optimization based on small molecule and condensed phase macromolecular target data. *J. Comp. Chem.*, **21,** 86-104.
11. Humphrey, W., Dalke, A. and Schulten, K. (1996) VMD-Visual Molecular Dynamics. *J. Mol. Graphics,* **14**, 33-38.
12. Prévost, C. and Takahashi, M. (2003) Geometry of the DNA strands within the RecA nucleofilament: role in homologous recombination. *Quart. Rev. Biophys.*, **36**, 429-453.
13. Prévost, C., Takahashi, M. and Lavery, R. (2009). Deforming DNA: from physics to biology. *ChemPhysChem.,* **10**, 1399-1404.
14. Ragunathan, K., Joo, C. and Ha, T. (2011) Real time observation of strand exchange reaction with high spatiotemporal resolution. *Structure,* **19**, 1064-1073.
15. van der Heijden, T., Modesti, M., Hage, S., Kanaar, R., Wyman, C. and Dekker, C. (2008) Homologous recombination in real time: DNA strand exchange by RecA. *Molecular Cell*, **30**, 530-538.
16. Peacock-Villada, A., Yang, D., Danilowicz, C., Feinstein, E., Pollock , N., Mc-Shan, S., Coljee, V.W. and Prentiss, M. (2012) Complementary strand relocation may play vital roles in RecA-based homology recognition. *Nucleic Acids Res.,* **40**, 10441-10451.

17. Hsieh, P., Camerini-Otero, C.S. and Camerini-Otero, R.D. (1992) The synapsis event in the homologous pairing of DNAs: RecA recognizes and pairs less than one helical repeat of DNA. *Proc. Natl Acad. Sci. U.S.A.*, **89**, 6492-6496.
18. Vlassakis, J., Feinstein, E., Yang, D., Tilloy, A., Weiller, D., Kates-Harbeck, J., Coljee, V. and Prentiss, M. (2013) Tension on dsDNA bound to ssDNA-RecA filaments may play an important role in driving efficient and accurate homology recognition and strand exchange. *Physical Review E*, **87**, 032702.
19. Danilowicz, C., Feinstein, E., Conover, A., Coljee, V.W., Vlassakis, J., Chan, Y.L., Bishop, D.K. and Prentiss, M. (2012) RecA homology search is promoted by mechanical stress along the scanned duplex DNA. *Nucleic Acids Res.*, **40**, 1717-1727.
20. Danilowicz, C., Limouse, C., Hatch, K., Conover, A., Coljee, V.W., Kleckner, N. and Prentiss, M. (2009) The structure of DNA overstretched from the 5'5' ends differs from the structure of DNA overstretched from the 3'3' ends. *Proc. Natl. Acad. Sci. U.S.A.*, **106**, 13196-13201.
